# Environmentally Friendly UV Absorbers: Synthetic Characterization and Biosecurity Studies of the Host–Guest Supramolecular Complex

**DOI:** 10.3390/ijms25158476

**Published:** 2024-08-03

**Authors:** Luwei Tian, Yanan Wu, Yetong Hou, Yaru Dong, Kaijie Ni, Ming Guo

**Affiliations:** College of Chemistry and Materials Engineering, Zhejiang Agriculture & Forestry University, Hangzhou 311300, China; tianluwei_vip@sina.com (L.T.); wuyanan0415@163.com (Y.W.); houyetong123@163.com (Y.H.); dongyaru1222@163.com (Y.D.)

**Keywords:** environmentally friendly UV absorbers, isoamyl 4-methoxycinnamate (IMC), sulfobutyl-β-cyclodextrin sodium salt (SBE-β-CD), biosecurity, AOS tree evaluation system

## Abstract

Isoamyl 4-methoxycinnamate (IMC) is widely used in various fields because of its exceptional UV-filter properties. However, due to its cytotoxicity and anti-microbial degradability, the potential eco-environmental toxicity of IMC has become a focus of attention. In this study, we propose a host–guest supramolecule approach to enhance the functionality of IMC, resulting in a more environmentally friendly and high-performance materials. Sulfobutyl-β-cyclodextrin sodium salt (SBE-β-CD) was used as the host molecule. IMC-SBE-β-CD supramolecular substances were prepared through the “saturated solution method”, and their properties and biosecurity were evaluated. Meanwhile, we conducted the AOS tree evaluation system that surpasses existing evaluation approaches based on apoptosis, oxidative stress system, and signaling pathways to investigate the toxicological mechanisms of IMC-SBE-β-CD within human hepatoma SMMC-7721 cells as model organisms. The AOS tree evaluation system aims to offer the comprehensive analysis of the cytotoxic effects of IMC-SBE-β-CD. Our findings showed that IMC-SBE-β-CD had an encapsulation rate of 84.45% and optimal stability at 30 °C. Further, IMC-SBE-β-CD promoted cell growth and reproduction without compromising the integrity of mitochondria and nucleus or disrupting oxidative stress and apoptosis-related pathways. Compared to IMC, IMC-SBE-β-CD is biologically safe and has improved water solubility with the UV absorption property maintained. Our study provides the foundation for the encapsulation of hydrophobic, low-toxicity organic compounds using cyclodextrins and offers valuable insights for future research in this field.

## 1. Introduction

Cinnamate UV absorbers (CUVAs), including isoamyl 4-methoxycinnamate (IMC), are widely used in various industries such as personal care products and polymer materials due to their exceptional ultraviolet absorption properties [1,2]. Despite this, our understanding of the potential toxicity of CUVAs is still limited. IMC, as the derivative of CUVAs, has been approved for use in personal care products such as sunscreens and shampoos in many countries [3]. However, research has indicated that IMC is photosensitive, which may lead to contact dermatitis upon skin contact [4,5]. Moreover, its resistance to degradation by microorganisms can result in bioaccumulation and adverse effects on human health [2]. IMC’s limited solubility in water and reliance on organic solvents further restrict its application. These challenges highlight the need to develop a more environmentally friendly and biocompatible version of IMC.

Supramolecular compounds are complex and organized chemical systems formed by non-valent bonding between the host and guest molecules involving interactions such as hydrogen bonds, hydrophobic forces, electrostatic interactions, van der Waals forces, π-π interactions, and other non-valent binding interactions [6]. Cyclodextrins (CDs), including sulfobutyl-β-cyclodextrin sodium salt (SBE-β-CD), are a type of macrocycle that can form supramolecular compounds [7]. CDs are cyclic oligosaccharides composed of D-glucose linked by α-1,4 glycosidic bonds and have a cavity structure with “hydrophobic inside and hydrophilic outside” characteristics [8]. The most common CDs can have 6, 7, or 8 glucopyranose units and are referred to as α-, β-, and γ-cyclodextrins (α-CD, β-CD, γ-CD), respectively. The depth of the cavities for the three CDs is the same (around 7.8 Å), while the cavity diameters of α-CD, β-CD, and γ-CD are 6, 8, and 10 Å, respectively [9]. SBE-β-CD is a critical member of the β-CD family that has the advantages of high stability, solubility in water, and biocompatibility [10,11]. It carries a negative charge, leading to an expansion of the internal cavity and an increase in the number of encapsulated guest molecules, thereby enhancing the solubility of the guest [11,12]. In addition, since SBE-β-CD is inherently non-toxic, it is reported to reduce the toxicity of guest molecule while improving biocompatibility [13].

**Figure 1:**
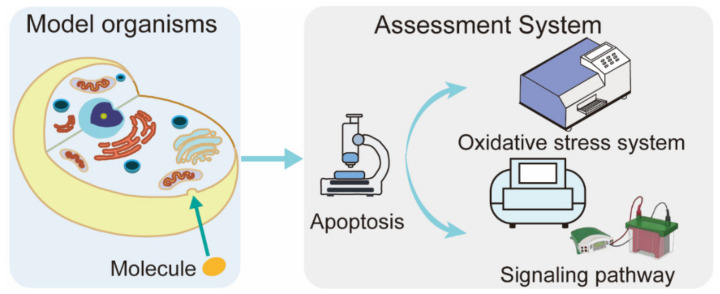
Diagram of AOS tree assessment system.

In this work, we propose a host–guest supramolecule approach using SBE-β-CD as the host molecule, aiming to enhance the functionality of IMC and result in a more environmentally friendly and high-performance materials. The preparation of inclusion complex was explored using the saturated solution method and solid phase synthesis method. The binding characteristics, functional groups, and crystal structures of IMC-SBE-β-CD inclusion complexes were characterized using techniques such as the phase solubility method, FT-IR method, and XRD method. It is worth noting that the current toxicological mechanisms of contaminants are often studied by single assays, which do not provide a comprehensive and objective understanding of the toxic effects and mechanisms of contaminants on cells. Therefore, it is vital to develop a method that reflects the overall toxicological mechanism of contaminants. Based on the theory of a heterogeneous network (PGCN) [14] mapped by genes, diseases, and individual trails, we established an AOS tree model for biosecurity assessment. As shown in Figure 1, the AOS tree system covers both model organisms and assessment systems. After a molecule acts on a cell, the molecule is subsequently evaluated by three aspects: apoptosis, oxidative stress system, and signaling pathway. This model, compared to a single assay, can offer a more comprehensive analysis of the cytotoxic effects of the prepared IMC-SBE-β-CD inclusion complex. This study provides a foundation for the encapsulation of hydrophobic, low-toxicity UV absorber compounds using cyclodextrins and offers a basis for further research on the ecological safety and health risks of these related compounds.

## 2. Results and Discussions

### 2.1. Standard Curves and the Inclusion Rate of IMC-SBE-β-CD

**Figure 2:**
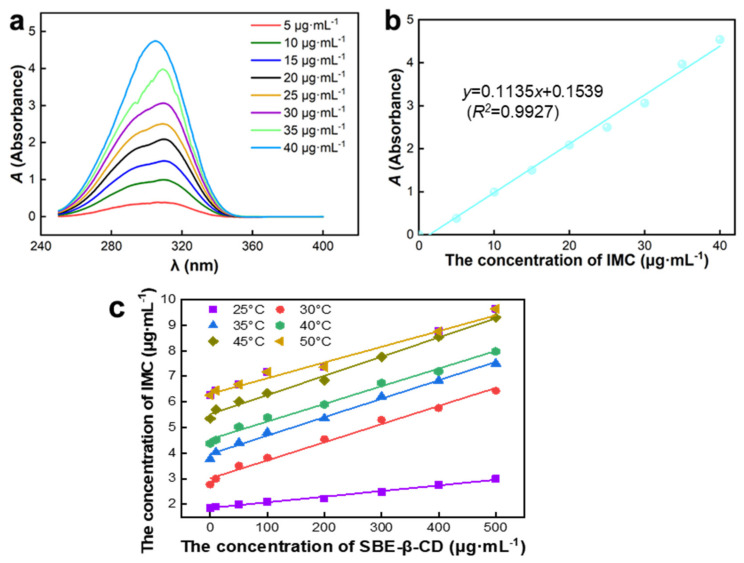
UV absorption spectra. (**a**) Spectra of IMC; (**b**) standard curve; (**c**) phase solubility curves and thermodynamic curves at different temperatures.

As shown in Figure 2a, the absorbance value at 310 nm increased with the increase in IMC concentration. The absorbance value and IMC concentration were positively correlated. From Figure 2b, the equation of IMC standard curve is *y* = 0.1135*x* + 0.1539 (*R*^2^ = 0.9927), which indicates that the IMC concentration has a good linear relationship with the absorbance value in a certain range.

The inclusion ratio is an important parameter to characterize the properties of inclusion complex, inclusion ratio can be calculated according to Equation (1):

(1) $$\mathrm{Inclusion}\;\mathrm{ratio}=\frac{\mathrm{IMC}\;\mathrm{concentration}\;\mathrm{in}\;\mathrm{inclusion}\;\mathrm{complex}}{\mathrm{Initial}\;\mathrm{IMC}\;\mathrm{concentration}}\times 100\%$$

The inclusion complex was analyzed by UV-vis absorption where the characteristic absorption peak of IMC was observed (Figure S1). The concentration of IMC in inclusion complex was calculated according to the equation of IMC standard curve. The results of the inclusion rates of inclusion compounds calculated according to Equation (1) are shown in Table 1, the IMC-SBE-β-CD inclusion rate was 84.45%, while the IMC/SBE-β-CD inclusion rate was 71.01%, indicating that the saturated solution method is more suitable for SBE-β-CD inclusion of IMC than the solid phase synthesis method.

As shown in Figure 2c, IMC solubility and SBE-β-CD concentration were positively and linearly correlated at the same temperature. This indicates that the solubility of IMC in SBE-β-CD shows an AL-type linear relationship. It also further indicates that IMC and SBE-β-CD are encapsulated with a 1:1 encapsulation ratio. The equations of the phase solubility curves of SBE-β-CD encapsulated IMC at different temperatures are shown in Table 2. When the mass concentration of SBE-β-CD was in the range of 0~500 μg·mL^−1^, the phase solubility curve had a good linear relationship. The K value of the binding constant of the inclusion property is an important parameter to measure the strength of the binding between the guest molecule and the subject molecule during the formation of the inclusion complex. The magnitude of the K value reflects the stability of the inclusion complex, i.e., the larger the K value, the more stable the inclusion complex is, and the higher the degree to which the guest molecule is wrapped by the subject molecule. In addition, the K-value is also related to the temperature, with the increase in temperature, the K-value increases, indicating that the inclusion is more and more stable; this is because the molecular thermal motion with the increase in temperature and becomes more intense, which favors the guest molecules to enter into the cavity of the subject molecules, and thus the reaction proceeds in the direction favorable to the encapsulation. The binding constant K can be calculated by the Higuchi–Connors Equation (2) [15]. It can be seen from Table 2 that the stability of the inclusion compound varies considerably by temperature. When the temperature is 30 °C, the best effect of SBE-β-CD encapsulated IMC, the best stability of IMC-SBE-β-CD was also obtained, and the stability decreases at higher temperatures.

(2) $$K=\frac{a}{b\;\times \;(1\;-\;a)}$$

where a is the slope of the phase solubility curve and b is the intercept of the phase solubility curve.

### 2.2. Quantitative Calculation of IMC-SBE-β-CD Optimized Conformation

SBE-β-CD molecule has the form of a truncated cone with the narrower side constituted by primary hydroxyls (primary side) and the wider side formed by secondary hydroxyls (secondary side), which makes SBE-β-CD “hydrophilic outside and hydrophobic inside” and can encapsulate hydrophobic guest molecules, thus improving the water solubility and stability of the guest molecules. There are four possible conformations of IMC encapsulated with SBE-β-CD (Figure S3). Methoxy entering from the secondary side of SBE-β-CD (Figure S3a), the ester group entering from the secondary side of SBE-β-CD (Figure S3b), methoxy entering from the primary side of SBE-β-CD (Figure S3c), and the ester group entering through the primary side of SBE-β-CD (Figure S3d).

In this experiment, the PM6 method in Gaussian 16 software was used to perform fully optimized SCF calculations for four IMC-SBE-β-CD conformations as well as the conformations where IMC is outside SBE-β-CD. The 3D structure of SBE-β-CD was constructed according to the reported literature [16]. The 2D structure of IMC was drawn in ChemDraw 20.0 and converted to 3D structure. SBE-β-CD and IMC were optimized using Gaussian 16. A structural guess of each host/guest complex was created with Gaussview 6 before being submitted for electronic structure calculation using the Gaussian 16 package [17]. Optimized structures, enthalpies, Gibbs energies, vibrational frequencies, and intensities are all computed. All energies reported are at 298 K and 1 atm with the unit kJ·mol^−1^. The energy values of the optimized complexes are summarized in Table S1. The binding energies of conformations where IMC is outside SBE-β-CD (416.0 kJ·mol^−1^ and 425.3 kJ·mol^−1^) are all lower than the inclusion complexes. The binding energy of configuration A is 476.3 kJ·mol^−1^, the binding energy of configuration B is 491.6 kJ·mol^−1^, the binding energy of configuration C is 484.2 kJ·mol^−1^, and the binding energy of conformation D is 498.9 kJ·mol^−1^. It can be seen that the ester group (-COO) of IMC guest molecules tend to enter the cavity. Both the IMC guest molecule methoxy (-OCH_3_) and ester group (-COO) in the four conformations formed hydrogen bonds with -OH of SBE-β-CD, and the binding of -COO to the primary side of SBE-β-CD showed the lowest conformer of the complex.

### 2.3. FT-IR and XRD Analysis of Materials

**Figure 3:**
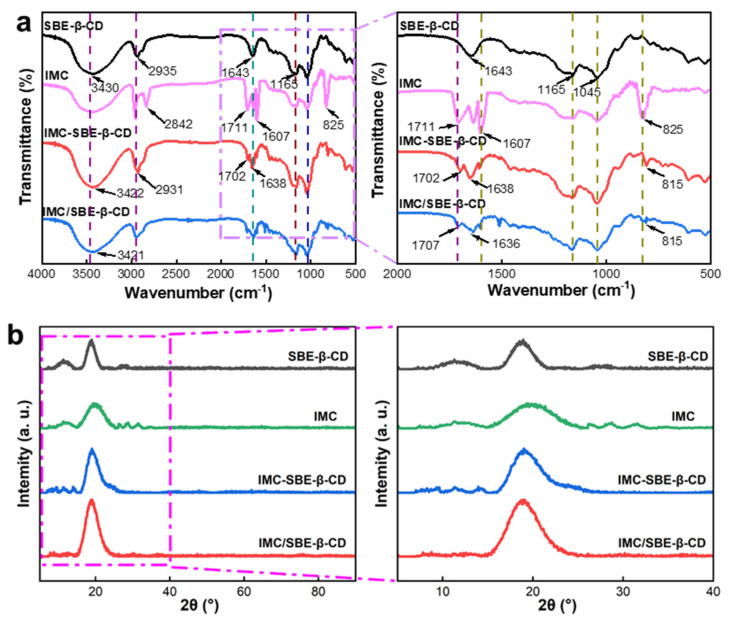
FT-IR and XRD analysis of IMC, SBE-β-CD, IMC-SBE-β-CD, and IMC/SBE-β-CD. (**a**) FT-IR analysis; (**b**) XRD analysis.

The structural property of IMC-SBE-β-CD was characterized by IR analysis (see Figure 3a). For comparison purposes, related starting materials were also examined by IR analysis. In the IR spectrum of SBE-β-CD, wide absorption bands were observed at 3430 cm^−1^ (O–H stretching), 2935 cm^−1^ (aliphatic C–H and CH_2_ stretching), 1643 cm^−1^ (H–O–H bending vibration of water molecule attached to the CD), 1165 cm^−1^ (C–O–C asymmetric stretching), and 1045 cm^−1^ (sulfoxide stretching) [18]. The typical peaks of IMC were observed at 2842 cm^−1^ (C–H stretching), 1711 cm^−1^ (C=O stretching), and 1607 cm^−1^ (stretching of benzene ring skeleton vibration), and the absorption peak at 825 cm^−1^ was an extremely strong p-disubstituted benzene, which is one of the characteristic peaks of IMC [19]. In the IR spectrum of IMC-SBE-β-CD, the intensity of characteristic peaks of IMC was weakened, and the absorption peaks at 3430 cm^−1^ and 1643 cm^−1^ of SBE-β-CD shifted to 3422 cm^−1^ and 1638 cm^−1^, respectively. Moreover, the absorption peaks of IMC at 1711 and 825 cm^−1^ shifted to 1702 and 815 cm^−1^, respectively. Similar results were also observed for IMC/SBE-β-CD. These observations suggest that IMC molecules were encapsulated into the cavity of SBE-β-CD by hydrophobic force, hydrogen bonds, and other secondary bonds and restrained by the cavity [20,21].

XRD was performed to investigate the physical form (crystalline or amorphous) of IMC, SBE-β-CD, and inclusion complexes. As shown in Figure 3b, IMC and SBE-β-CD show broad characteristic diffraction peaks in the scan range, which concludes that both IMC and SBE-β-CD are in amorphous states. IMC-SBE-β-CD and IMC/SBE-β-CD show broad diffraction peaks but not a superposition of IMC and SBE-β-CD diffraction patterns. In addition, the diffraction peak of IMC in IMC-SBE-β-CD and IMC/SBE-β-CD significantly reduced or disappeared. These observations suggest the formation of the inclusion complex, in which IMC molecule binds with SBE-β-CD in the cavity by hydrophobic force, van der Waals force, and other forces [22].

### 2.4. Cell Viability and Morphology

Previous studies conducted by our research group have elucidated the disruptive effects of IMC on cellular functions through various pathways, including gene expression, protein expression, and cell structure and function. These disruptions lead to a decrease in mitochondrial membrane potential, an increase in ROS levels, and ultimately the initiation of early apoptosis [23]. Building on this knowledge, the properties and biosecurity of the prepared inclusion complex were evaluated in detail.

**Figure 4:**
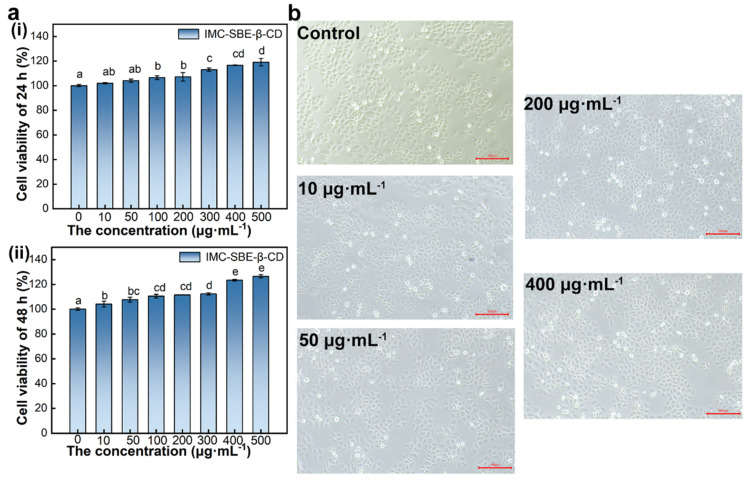
The effect of IMC-SBE-β-CD on cell viability and morphology. (**a**) The effect of IMC-SBE-β-CD on cell viability (lowercase letters denote *p* < 0.05 as obtained using one-way analysis of variance) ((**i**) 24 h, (**ii**) 48 h); (**b**) the effect of IMC-SBE-β-CD on cell morphology (scale bar: 100 μm).

SMMC-7721, a hepatocellular carcinoma cell line commonly used in the laboratory, does not mimic the use of sunscreen, but was chosen to be more representative of the toxicological effects of chemicals in sunscreen as they enter the body through the water cycle and are metabolized in the liver. As shown in Figure 4a, cell viability was positively correlated with the IMC-SBE-β-CD concentration and duration of action. Cell viability after intervention with different concentrations of IMC-SBE-β-CD was higher than that of the control group, indicating that IMC-SBE-β-CD can promote cell growth and reproduction. In the range of 10~200 μg·mL^−1^, IMC-SBE-β-CD promoted cell reproduction and growth at a slower rate, while in the range of 300~500 μg·mL^−1^, IMC-SBE-β-CD promoted cell reproduction and growth at a faster rate. When IMC-SBE-β-CD intervened in SMMC-7721 cells for 24 h, the difference was significant between 0 μg·mL^−1^, 300 μg·mL^−1^, and 500 μg·mL^−1^ experimental groups. When IMC-SBE-β-CD intervened for 48 h, the difference was significant between the 0 μg·mL^−1^, 10 μg·mL^−1^, and 300 μg·mL^−1^ experimental groups. The original study of the group showed that the cell viability decreased in a dose-dependent and intervention time-dependent manner [23]. And cell viability increased after IMC-SBE-β-CD intervention, indicating that IMC-SBE-β-CD promotes cell growth and reproduction. In order to compare and analyze the cytotoxicity of IMC and IMC-SBE-β-CD, five concentrations of 0 μg·mL^−1^, 10 μg·mL^−1^, 50 μg·mL^−1^, 200 μg·mL^−1^, and 400 μg·mL^−1^ were selected as intervention conditions for SMMC-7721 cells for 24 h, according to the original study of the group [23].

Because SMMC-7721 cells can proliferate indefinitely when cultured in vitro with adequate nutrition as well as similar to normal stem cells in terms of oxidative stress and apoptosis [24], SMMC-7721 cells are an ideal in vitro cell model for studying the toxicity and mechanism of action of ultraviolet absorbers (UVAs) on human cells. Cell morphology is diverse, and similar cells in different states behave differently. The effect of UVAs on cell morphology can be grasped visually by observing cell morphology changes through light microscopy. From Figure 4b, it can be clearly observed that the cells in the IMC-SBE-β-CD experimental group showed epithelial cell morphology, mostly spindle-shaped, and the majority of cells were in logarithmic growth phase. The cells in experimental groups had a full morphology with clear visible contours, tightly connected between cells and vigorous growth. As the concentration of IMC-SBE-β-CD increased, the cell density increased, indicating that IMC-SBE-β-CD has a promotional effect on the normal growth and reproduction of SMMC-7721 cells. As the concentration of IMC-SBE-β-CD increased, the number of cell proliferation increased.

### 2.5. Effect of IMC-SBE-β-CD on Apoptosis

In normal cells, the cell membrane is selectively permeable, preventing harmful substances or substances that the cell itself does not need from entering the cell. When cells are subjected to external stress, cell membrane permeability is enhanced and harmful substances can freely enter the cells. Hoechst 33342 is a blue fluorescent dye, and a small amount of Hoechst 33342 is able to pass through the cell membrane into the cell in its normal state, while when the cell is damaged, Hoechst 33342 enters the cell in large quantities through the cell membrane, resulting in enhanced blue fluorescence inside the cell. As shown in Figure S2a,d(i), for the IMC-SBE-β-CD experimental group, Hoechst 33342 fluorescence staining resulted in uniform cell fluorescence, large and full nuclei, and smooth nuclear membranes with a dark blue distribution, indicating that IMC-SBE-β-CD did not cause toxic on the cells. The quantitative analysis of cell fluorescence intensity after Hoechst 33342 staining revealed a decreasing trend of cell fluorescence intensity in the IMC-SBE-β-CD experimental group, and there was a significant difference in fluorescence intensity between the experimental groups.

ROS are a group of oxygen-containing compounds that are mainly produced in mitochondria, endoplasmic reticulum, and peroxisomes. At the same time, cytokines, free fatty acids, and other factors can also trigger the production of ROS. The production of ROS in cells is in a dynamic balance, and a small amount of ROS can promote cell proliferation and differentiation. When the cells are stimulated or stressed by external factors, the dynamic balance is broken and the accumulation of ROS can cause oxidative stress damage to the cells, leading to apoptosis. ROS play an essential role in cell signaling and homeostasis in vivo. When cells are subjected to external stress, intracellular ROS levels rise, severely affecting cell structure and function. As shown in Figure S2b, the green fluorescence intensity of control cells stained by H_2_DCFDA probe was weaker, indicating that the intracellular ROS level was lower and the cells were in good condition at this time. Intracellular green fluorescence was reduced after IMC-SBE-β-CD intervention. The quantitative analysis of ROS fluorescence intensity by Image J software v1.8.0-112 showed that the ROS level gradually decreased with the increase in IMC-SBE-β-CD concentration, that the difference was not significant between the experimental groups at high concentrations (200 μg·mL^−1^ and 400 μg·mL^−1^), and that significant differences existed between the remaining experimental groups.

Mitochondria are intracellular organelles that convert energy and control apoptosis. When cells are subjected to external stress, changes in mitochondrial membrane potential represent an early apoptotic state. The JC-1 probe, a novel fluorescent probe for mitochondrial membrane potential determination, can be present in the cell as aggregates and monomers, resulting in red and green fluorescence, respectively. JC-1 is a fluorescent probe that can rapidly and sensitively detect changes in mitochondrial membrane potential in cells, tissues, or blunted mitochondria and can be used for early apoptosis detection. When the mitochondrial membrane potential is high, JC-1 aggregates in the mitochondrial matrix, forming a polymer, which can produce red fluorescence; when the mitochondrial membrane potential is low, JC-1 cannot be aggregated in the mitochondrial matrix, and then JC-1 is a monomer, which can produce green fluorescence. In this way, it is very convenient to detect the change in cellular mitochondrial membrane potential through the change in fluorescence color. The relative proportion of red and green fluorescence is commonly used to measure the proportion of mitochondrial depolarization. Changes in mitochondrial membrane potential are a hallmark event in the early stages of apoptosis. As shown in Figure S2c, with the increase in IMC-SBE-β-CD concentration, the green fluorescence of cells in the experimental group gradually decreased, the red fluorescence was significantly enhanced, and the number of cells within the field of view increased. When concentration (Figure S2a) of IMC-SBE-β-CD was 400 μg·mL^−1^, the cells within the field of view were predominantly red fluorescent, with the least number of green fluorescent cells. As seen in Figure S2c, the relative intensity of red/green fluorescence increased with the increase in IMC-SBE-β-CD concentration, indicating that the mitochondrial membrane potential of the cells increased after IMC-SBE-β-CD intervention and no apoptosis occurred.

### 2.6. Effect of IMC-SBE-β-CD on Oxidative Stress System

**Figure 5:**
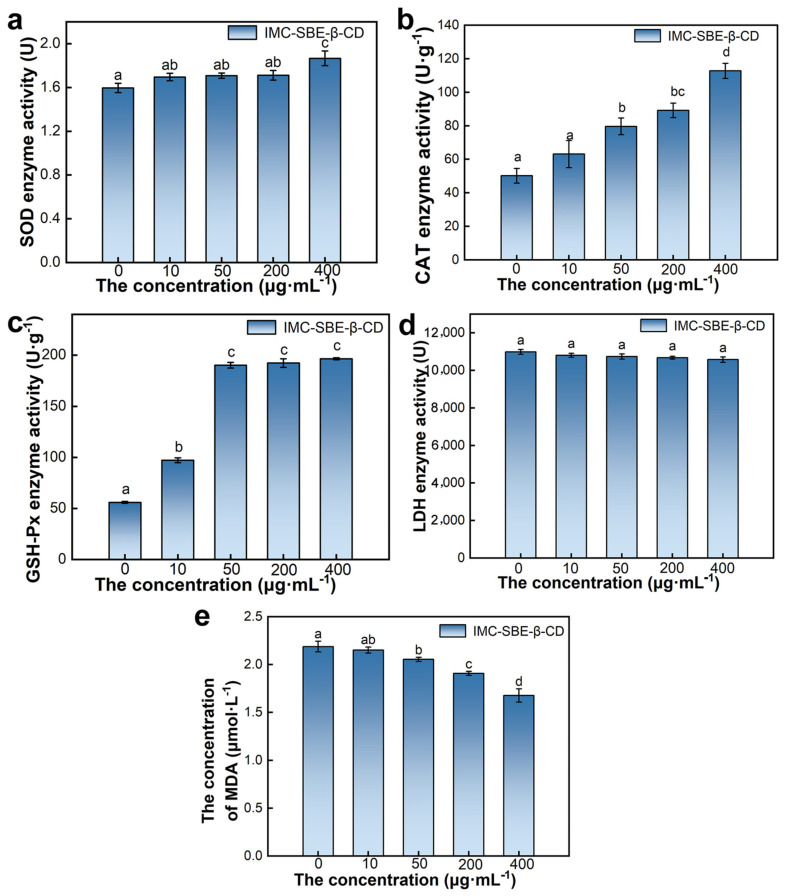
Effect on oxidative stress systems (lowercase letters denote *p* < 0.05). (**a**) SOD; (**b**) CAT; (**c**) GSH-Px; (**d**) LDH; (**e**) MDA.

Oxidative stress occurs due to the overactivity of oxidative enzymes or dysfunction of the antioxidant enzyme system [25]. When the cell is in an adverse environment, a large number of oxygen radicals are present in the cell, disrupting the balance of free radical dynamics and attacking the intracellular membrane structure, resulting in an enhanced degree of lipid peroxidation. At this time, antioxidant enzymes such as superoxide dismutase (SOD), catalase (CAT), and glutathione peroxidase (GSH-Px) are produced in the cell to reduce the content of oxygen radicals in the cell and keep them in dynamic balance [26]. Since malondialdehyde (MDA) is one of the most important products of lipid peroxidation in cellular models, its content can also be measured to reflect the effect of external stress on the degree of oxidative stress in cells. Lactate dehydrogenase (LDH) is normally present in the cytoplasm, and after external stress, the cell membrane structure is disrupted and LDH will flow out of the cell, so the measurement of LDH activity in the culture medium can indirectly reflect the degree of cell damage. As shown in Figure 5, as the concentration of IMC-SBE-β-CD increased, the enzyme activity of SOD, GSH-Px, and CAT increased; the content of MDA decreased; and the enzyme activity of LDH enzyme activity decreased. When the concentration of IMC-SBE-β-CD was 400 μg·mL^−1^, the enzyme activity of SOD and CAT, and the content of MDA were significantly different between the other experimental groups. There was no significant difference in LDH enzyme activity between the experimental groups with different concentrations of IMC-SBE-β-CD, and the difference in GSH-Px enzyme activity between the experimental groups with medium and high concentrations of IMC-SBE-β-CD (50 μg·mL^−1^, 200 μg·mL^−1^, and 400 μg·mL^−1^) was not significant.

### 2.7. Effect of IMC-SBE-β-CD on Apoptotic Gene Expression

**Figure 6:**
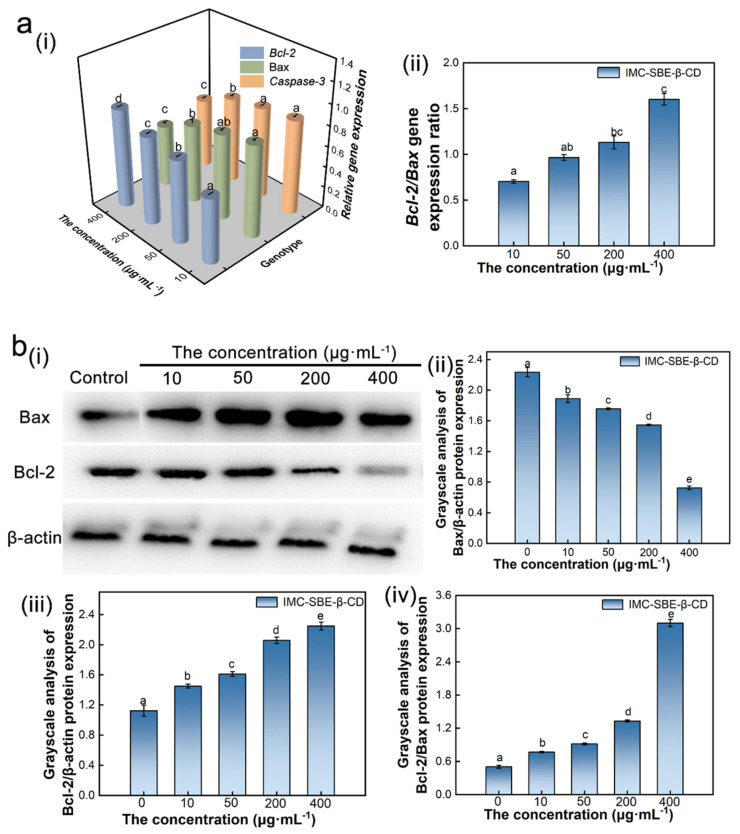
Effect of IMC-SBE-β-CD on gene expression and protein expression: (**a**) gene expression ((**i**) relative expression of genes, (**ii**) expression ratio of Bcl-2/Bax genes); (**b**) protein expression ((**i**) protein bar diagram, (**ii**) Bax protein grayscale analysis, (**iii**) Bcl-2 protein grayscale analysis, (**iv**) Bcl-2/Bax protein greyscale ratio analysis). Lowercase letters denote *p* < 0.05.

Normal gene expression is the basis for cells to maintain normal physiological activities. When gene expression is disrupted, the synthesis and secretion of intracellular proteins will also be affected, leaving the cell structure disabled and eventually leading to apoptosis. The mitochondrial apoptotic pathway, as one of the important pathways of apoptosis process, can control apoptosis by changing the structure and function of mitochondrial membrane and releasing apoptotic factors [27]. As shown in Figure 6a, with the increase in IMC-SBE-β-CD concentration, the expression of Bax and Caspase-3 mRNA down-regulated, while Bcl-2 mRNA expression up-regulated. There were significant differences in Bcl-2 mRNA expression in the experimental groups with different concentrations of IMC-SBE-β-CD. Significant differences in Caspase-3 mRNA expression were found in the experimental groups with high concentrations (200 μg·mL^−1^, 400 μg·mL^−1^), while the differences in Caspase-3 mRNA expression in the remaining experimental groups were not significant. For Bax mRNA, there was a significant difference between the experimental groups except for the concentration of 50 μg·mL^−1^. The Bcl-2/Bax mRNA ratio increased with the increase in IMC-SBE-β-CD concentration. The Bcl-2/Bax mRNA ratio between the experimental group with high concentration (400 μg·mL^−1^) and the experimental group with 10 μg·mL^−1^ was statistically significant.

Protein is one of the macromolecules in living organisms and plays an important role in biological functions. The biological functions of proteins mainly include signal transduction, gene transcription, apoptosis, immune function, catalytic function, structural proteins, etc. [28]. It has been found that certain gene products (proteins or enzymes) associated with apoptosis can be localized in mitochondria, altering mitochondrial membrane permeability and leading to apoptosis. Among them, Bcl-2 family proteins can directly alter mitochondrial permeability and lead to apoptosis [29]. From Figure 6b(i), it can be seen that the expression of the internal reference protein β-actin in the experimental groups with different concentrations of IMC-SBE-β-CD is consistent, indicating that the protein samples of WB have the same spot sample content. The gray-scale analysis of the protein bands showed that with the increase in IMC-SBE-β-CD concentration, the Bax protein expression increased, and conversely, the Bcl-2 protein expression decreased and the Bcl-2/Bax protein expression ratio increased. Meanwhile, Bax and Bcl-2 protein expression as well as Bcl-2/Bax protein expression ratio was significantly different between the experimental groups at different concentrations.

## 3. Materials and Methods

### 3.1. Materials and Equipment

IMC (>95%) was purchased from Tokyo Chemical Industry (Tokyo, Japan). SBE-β-CD (>97%) was purchased from McLean Biotechnology Co., Ltd. (Shanghai, China). The human hepatoma cell line SMMC-7721 was purchased from the Library of Tumor Cells of the Chinese Academy of Medical Sciences (Beijing, China). The Supplementary Materials contains further information about materials and equipment required for the experiments and solution preparation.

### 3.2. Saturated Solution Method

IMC-SBE-β-CD: IMC solution (0.1 mol·L^−1^) was prepared in anhydrous ethanol and SBE-β-CD solution (0.1 M) was prepared in double distilled water (ddH_2_O). The same volume of IMC solution and SBE-β-CD solution were mixed and stirred at room temperature for 72 h, then centrifuged and freeze-dried to obtain the IMC-SBE-β-CD inclusion complex. The product was washed with ethanol to ensure complete removal of residual IMC.

### 3.3. Solid-Phase Synthesis Method

IMC/SBE-β-CD: This inclusion complex was obtained by weighing and grinding in a mortar with an SBE-β-CD/IMC ratio of 1:1. The product was washed with ethanol to ensure the complete removal of residual IMC.

### 3.4. Characterization of Supramolecular Inclusion Complex

The UV absorption method was used to determine the encapsulation properties of IMC-SBE-β-CD and IMC/SBE-β-CD. The quantum chemistry (QC) method was used to calculate the IMC and SBE-β-CD inclusion properties. Phase solubility method was used to calculate the binding constant *K*. Functional group changes in IMC-SBE-β-CD and IMC/SBE-β-CD were determined by Fourier infrared spectroscopy (FT-IR), and the crystal changes in IMC-SBE-β-CD and IMC/SBE-β-CD were determined by X-ray diffraction (XRD). Supplementary Materials contains further information about the characterization of supramolecular inclusion complex.

### 3.5. CCK-8 Assay

CCK-8 is a rapid and highly sensitive assay for cell proliferation and cytotoxicity based on WST-8, an MTT-like compound that can be reduced in the presence of the electronically coupled reagent 1-Methoxy PMS to form yellow, water-soluble Formazan. The more proliferative the cell, the darker the color; the more cytotoxic the cell, the lighter the color (lower absorbance at 450 nm). The cells were exposed to IMC-SBE-β-CD working solutions for 24 h and 48 h. CCK-8 reagent was added, and the plates were incubated for 0.5 h. Cell viability was calculated by measuring the absorbance at 450 nm. All specific details about cell culture and CCK-8 assay can be found in the SI.

### 3.6. Cytomorphology Analysis

After exposure of the cells to the appropriate concentrations of IMC-SBE-β-CD for an appropriate time, according to the CCK-8 assay, cell morphology was observed by an inverted microscope (100×).

### 3.7. Apoptosis Assay

According to the manufacturer’s instructions, the cells of different groups were stained with the Hoechst 33342 staining solution, observed under an inverted fluorescent microscope.

### 3.8. ROS Assay

According to the instructions of the ROS assay kit, the H_2_DCFDA working solution (10 μmol·L^−1^) was used for the staining and observed under inverted fluorescence microscope.

### 3.9. Mitochondrial Membrane Potential (MMP) Assay

According to manufacturer’s instructions, the JC-1 working solution was used for the staining and observed under an inverted fluorescent microscope.

### 3.10. Measuring the Activity of Antioxidant Enzymes and the Content of Active Substance

After the cells were exposed to IMC-SBE-β-CD, the culture was collected in the Eppendorf tubes and centrifuged to collect the supernatant and then used to determine lactate dehydrogenase (LDH) enzyme activity. The cell lysate added RIPA lysis buffer was collected by refrigerated centrifugation and then used to determine the enzyme activities of Catalase (CAT), Superoxide dismutase (SOD), and Glutathione peroxidase (GSH-Px) and Monochrome display adapter (MDA) content. All specific details about measuring the activity of SOD, CAT, GSH-Px, and LDH and measuring the content of MDA can be found in the Supplementary Materials.

### 3.11. Evaluation of Gene Expression

The total RNA was extracted from cells using the AxyPrep (Axygen, San Francisco, CA, USA) total RNA miniprep kit according to the manufacturer’s instructions. cDNA was obtained by reverse transcription of RNA, and then amplified by PCR. All the primers used in this study are shown in Table S2. The 2^−∆∆Ct^ method was used to calculate the relative expression of *Bax* mRNA and Bcl-2 mRNA using *β-actin* mRNA as the internal reference.

### 3.12. Evaluation of Protein Expression

After the cells were treated, the lysis buffer was extracted and diluted with the aim of obtaining cell lysate. The lysate was subjected to 12% SDS-polyacrylamide gel electrophoresis (SDS-PAGE). Electrophoresis was followed by membrane transfer. Incubation with different antibodies was followed by visualization with ChemiDoc^TM^ XRS (BIO-RAD, Hercules, CA, USA).

## 4. Conclusions

In this study, we successfully developed an environmentally friendly material with high value using SBE-β-CD as the host molecule and IMC as the guest molecule. The IMC-SBE-β-CD not only retains the UV-absorbing properties of IMC but also exhibits improved bio-security and enhanced water solubility. We introduced the AOS tree assessment system, showing that IMC-SBE-β-CD promoted cell growth and reproduction without causing damage to organelles such as the nucleus and mitochondria. The intracellular ROS level decreased, while the activities of antioxidant enzymes such as SOD, CAT, and GSH-Px increased after IMC-SBE-β-CD intervention. Additionally, the activity of LDH enzyme and the content of MDA decreased, indicating that IMC-SBE-β-CD did not disrupt intracellular redox homeostasis and cause cell damage. The down-regulation of *Bax* mRNA and Caspase-3 mRNA, as well as the up-regulation of Bcl-2 mRNA expression, were observed after IMC-SBE-β-CD intervention. Furthermore, Bax protein expression increased, while Bcl-2 protein expression decreased, further indicating that IMC-SBE-β-CD did not have toxic effects on cells. This work can provide an experimental and theoretical basis for the application of cyclodextrin-encapsulated hydrophobic and low-toxicity organics as biocompatible materials in various fields.

## Figures and Tables

**Table 1:** Inclusion rates of IMC-SBE-β-CD and IMC/SBE-β-CD.

Materials	IMC Concentration in Inclusion Complex (μg·mL^−1^)	Inclusion Ratio (%)
IMC-SBE-β-CD	10.48	84.45
IMC/SBE-β-CD	8.82	71.01

**Table 2:** Phase solubility curves and binding constants K values for SBE-β-CD-encapsulated IMC.

Temperature (°C)	Phase Solubility Curve	*R* ^2^	K Values (L·mmol^−1^)
25	*y* = 0.0022*x* + 1.8569	0.9881	1.1874
30	*y* = 0.0071*x* + 3.0122	0.9853	2.3739
35	*y* = 0.0072*x* + 3.9635	0.9935	1.8298
40	*y* = 0.0069*x* + 4.5450	0.9890	1.5287
45	*y* = 0.0075*x* + 5.5185	0.9923	1.3693
50	*y* = 0.0063*x* + 6.3348	0.9859	1.0008

## Data Availability

Data is contained within the article or Supplementary Materials.

## Supplementary Materials

The following supporting information can be downloaded at: https://www.mdpi.com/article/10.3390/ijms25158476/s1.
