# Supplementary material for: Environmentally Friendly UV Absorbers: Synthetic Characterization and Biosecurity Studies of the Host–Guest Supramolecular Complex
Source: Int J Mol Sci. 2024 Aug 3;25(15):8476. doi: 10.3390/ijms25158476 (PMC11312980; doi:10.3390/ijms25158476)
Supplement: Supplementary file 1 [file ijms-25-08476-s001.zip › ijms-3122669-supplementary.pdf]

Environmentally Friendly UV Absorbers: Synthetic Characterization and Biosecurity Studies of the Host–Guest Supramolecular Complex

Luwei Tian, Yanan Wu, Yetong Hou, Yaru Dong, Kaijie Ni * and Ming Guo *

College of Chemistry and Materials Engineering, Zhejiang Agriculture & Forestry University, Hangzhou 311300, China; tianluwei_vip@sina.com (L.T.); wuyanan0415@163.com (Y.W.); houyetong123@163.com (Y.H.); dongyaru1222@163.com (Y.D.)

* Correspondence: nikaijie@zafu.edu.cn (K.N.); guoming@zafu.edu.cn (M.G.)

2. Materials and Methods

2.1. Materials and equipment

Disodium hydrogen phosphate, sodium dihydrogen phosphate, and anhydrous ethanol (AR grade) were purchased from Sinopharm Chemical Reagent Co., Ltd. (Beijing, China). Hoechst 33342 staining solution (100×) was purchased from Beyotime Biotechnology (Shanghai, China). Cell counting kit-8, reactive oxygen species (ROS) assay kit, mitochondrial membrane potential (MMP) detection kit (JC-1), fluorescent quantitative PCR kit (SYBR green master mix), BCA protein concentration assay kit, and total superoxide dismutase (SOD) assay kit with nitro-tetrazolium blue chloride (NBT) were purchased from Biosharp Co., (Beijing, China). Glutathione peroxidase (GSH-Px) assay kit, malondialdehyde (MDA) assay kit, lactate dehydrogenase (LDH) assay kit and catalase (CAT) assay kit were purchased from Leagene Biotechnology Co., Ltd. (Beijing, China). AxyPrep total RNA miniprep kit was purchased from Axygen (New York, USA). PrimeScript™ RT reagent Kit (Perfect Real Time) was purchased from Takara (Kyoto, Japan). Mouse anti-Bcl-2 monoclonal antibody and mouse anti-β-actin monoclonal antibody were purchased from Applygen Technologies Inc. (Beijing, China). Rabbit anti-Bax monoclonal antibody was purchased from Proteintech Group Inc (Chicago, USA). HRP-labelled goat anti-mouse lgG and HRP-labeled goat anti-rabbit lgG were purchased from Sangon Biotech Co., Ltd (Shanghai, China). Modified RMPI-1640 medium, fetal bovine serum (FBS), phosphate-buffered saline (PBS) (1×), penicillin-streptomycin solution, 0.25% trypsin solution (1×) were purchased from Cytiva (Logan, UT, USA). Other reagents are analytical grade.

The following equipment was used in this study: FA1004B electronic analytical balance (Youke Instruments Co., Ltd., Shanghai, China), DF-2000 collector magnetic stirrer (Geng Yu Instruments Co., Ltd., Hangzhou, China), SJLA-10N freeze dryer (Shuangjia Instruments Co., Ltd., Ningbo, China), UV-2550 ultraviolet (UV) spectrophotometer (Shimadzu Corporation, Kyoto, Japan), SU8010 cold field

emission scanning electron microscope (Hitachi High-Technologies, Tokyo, Japan), S20071649 fourier infrared spectroscopy (FT-IR) spectrometer (Thermo Fisher Scietific, Massachusetts, USA), XRD-6000 X-ray diffractometer (Shimadzu, Kyoto, Japan), ZD-9560 decolorization shaker oscillator (Kuncheng Ltd., Shanghai, China), HCB-1300V vertical laminar flow clean bench (Haier, Qingdao, China), Thermo DH-800 carbon dioxide culture box (Thermo Fisher Scientific, Massachusetts, USA), CKX31 inverted biological microscope (Olympus, Kyoto, Japan), TI-S fluorescent inverted microscope (Nikon, Tokyo, Japan), iMark 18067 microplate reader (Bio-Rad, Hercules, CA, USA), high-speed refrigerated centrifuge (Eppendorf, Hamburg, Germany), ChemiDoc™ XRS System (Bio-Rad, Hercules, CA, USA), CFX96™ Touch Real-Time PCR Detection System (Bio-Rad, Hercules, CA, USA), electrophoresis instrument and electrophoresis tank (Bio-Rad, Hercules, CA, USA), micropipette (Eppendorf, Hamburg, Germany)

2.2. Solution preparation

Na_2HPO_4 and NaH_2PO_4 were accurately weighed and dissolved in double-distilled water (ddH_2O) to prepare Na_2HPO_4 solution and NaH_2PO_4 solution (0.2 mol·L^{-1} (M)). Accurate measurement of Na_2HPO_4 solution and NaH_2PO_4 solution were mixed to obtain phosphate (PBS) buffer (0.2 M, pH 7.4).

Complete medium was prepared from 10% FBS, 100 international unit·mL^{-1} (IU·mL^{-1}) penicillin, 100 μg·mL^{-1} streptomycin, and RIPM-1640 medium.

2.3. Encapsulation performance

The mass concentrations of IMC solutions (5 μg·mL^{-1}, 10 μg·mL^{-1}, 15 μg·mL^{-1}, 20 μg·mL^{-1}, 25 μg·mL^{-1}, 30 μg·mL^{-1}, and 40 μg·mL^{-1}) were prepared in anhydrous ethanol. The spectra were measured from 250~400 nm using the UV spectrophotometer. The absorbance at 310 nm was extracted and the standard curve of mass concentration (c) ~ UV absorbance (A) of IMC was plotted.

The data were fitted to obtain a linear regression equation.

PBS buffer was prepared to obtain IMC-SBE-β-CD solution and IMC/SBE-β-CD solution (500 μg·mL^{-1}), and the linear regression equation of IMC was used to calculate the encapsulation rate and analyze the encapsulation performance.

2.4. Quantitative calculation of the inclusion configuration of supramolecular inclusion complexes

Quantum chemistry (QC) method was used to calculate the IMC and SBE-β-CD inclusion properties. Firstly, theoretical calculations were carried out to obtain the monomer-dominant configurations of IMC and SBE-β-CD, and the IMC-SBE-β-CD inclusion configurations were constructed and optimized to obtain the most stable configurations under various inclusion modes. The optimized calculations of the inclusion configurations were carried out using the PM6 approach without any symmetry constraints up to the energy minimum. All the computational work was carried out using Gaussian 09W software to quantitatively calculate the binding energies of IMC-SBE-β-CD inclusion complexes for each configuration and to analyze the most stable configuration.

2.5. Phase solubility method

A series of SBE-β-CD solutions (50 μg·mL^{-1}, 100 μg·mL^{-1}, 200 μg·mL^{-1}, 300 μg·mL^{-1}, 400 μg·mL^{-1}, 500 μg·mL^{-1}) were prepared in PBS buffer. An excess of IMC was added, and the solution was shaken at a constant temperature for 12 h at 25°C, 30°C, 35°C, 40°C, and 45°C, aimed to reach the dissolution equilibrium. Since IMC is insoluble in water, resulting in its deposition at the bottom of the solution, the supernatant was filtered through 0.45 μm microporous filter membrane to remove the undissolved IMC. The absorbance value was measured at 310 nm, and was brought into a linear regression equation to obtain the dissolved concentration of IMC. The phase solubility curve was plotted with the mass concentration of SBE-β-CD as the horizontal coordinate and the dissolved concentration of IMC as the vertical coordinate, and the phase solubility curve equation was

obtained by fitting it linearly and the binding constant *K* was calculated.

2.6. Fourier infrared spectroscopy (FT-IR)

The sample was ground and mixed according to the ratio of sample to KBr of 1:100 for pressing. The samples were placed on the sample holder of the FT-IR instrument and the wavelength range was set from 4000 cm^{-1} to 500 cm^{-1}.

2.7. X-ray diffraction (XRD)

The samples were well ground and spread as evenly as possible into the sample making frame, pressed and excess sample removed. The samples were placed on the sample stage of the XRD instrument, and the test conditions were set as X-ray source of Cu target K_{α} rays, voltage of 36 kV, current of 20 mA, scanning range of 5°~90°, and scanning speed of 2°·min^{-1}.

2.8. Cell culture

500 mg·mL^{-1} stock solutions of IMC-SBE-β-CD were prepared in ddH_2O, filtered through the 0.22 μm microporous filter to remove bacteria, and stored at -20°C without light. The working solutions of IMC-SBE-β-CD at concentrations of 10, 50, 100, 200, 300, 400, and 500 μg·mL^{-1} were set, and stored at 4°C protected from light.

SMMC-7721 cells were cultured in a complete medium. The cells were incubated in a 37°C, 5% (V/V) CO_2 incubator. The cells were washed with PBS, digested with 0.25% trypsin-EDTA digest and passaged at 70-80% confluence.

2.9. CCK-8 assay

Cell-free complete medium was used as blank control groups. Cells of experimental control groups were cultured in a complete medium. CCK-8 reagent was added, and the plates were incubated for 0.5 h. Cell viability was calculated according to equation (S1) by measuring the absorbance at 450 nm using the microplate reader.

$$\text{Cell viability}=\frac{OD_{\text{Experimental group}}-OD_{\text{Blank control group}}}{OD_{\text{Experimental control group}}-OD_{\text{Blank control group}}}\times 100\% \quad \text{(S1)}$$

Where OD values are the absorbance values of the different groups.

2.10 Apoptosis assay

Hoechst 33342: Cells were inoculated into 12-well cell culture plates at a density of 2.0×105 cells-mL-1. After the cells were adhered to the wall, the old medium was discarded, and medium containing different concentrations of IMC-SBE-β-CD or IMC/SBE-β-CD was added, and incubated in an incubator for 24 h. The medium was discarded, washed with PBS, and added with Hoechst 33342 staining solution, incubate in the incubator under light protection for 30 min. discard the staining solution, wash with PBS buffer for 3 times, and place under fluorescence microscope for observation and photographing.

2.11 ROS assay

ROS: The steps of cell spreading and drug intervention were the same as above. Configure H2DCFDA staining working solution (10 μmol-L-1 (μM)) according to the kit instructions. Configure H2DCFDA staining working solution (10 μM) according to the ratio of H2DCFDA stock solution (10 mmol-L-1 (mM)): PBS buffer = 1:1000, mix well and prepare for use. After cell intervention, discard the medium, wash with PBS, add H2DCFDA staining working solution and incubate in the incubator under light for 30 min. discard the staining working solution, wash with PBS for 2-3 times, and place under the microscope for observation and photographing.

2.12 Mitochondrial membrane potential (MMP) assay

JC-1: The steps of cell spreading and drug intervention were the same as above. Configure JC-1 staining working solution and staining buffer according to the instructions of the kit. After washing the adherent cells with PBS, add JC-1 staining working solution and incubate at 37℃ away from light for 20 min. Discard JC-1 staining working solution, wash with JC-1 staining buffer for 2 times, add PBS and observe under the fluorescence microscope for photographs.

2.13. Measuring the activity of SOD

The NBT color reaction was used to measure SOD enzyme activity. The cell lysate was added to the experimental groups. Control group 1 contained SOD buffer and reaction starter solution. SOD buffer was only added to the control groups 2. The plates were incubated at 37°C for 30 min. The absorbance values were measured at 562 nm, and the SOD enzyme activity was calculated according to equation (S2).

$$\text{SOD activity (U)}=\frac{\frac{OD_{\text{Experimental group}}-OD_{\text{Control group 1}}}{OD_{\text{Control group 1}}-OD_{\text{Control group 2}}}\times 100\%}{1-\left(\frac{OD_{\text{Experimental group}}-OD_{\text{Control group 1}}}{OD_{\text{Control group 1}}-OD_{\text{Control group 1}}}\times 100\%\right)} \quad \text{(S2)}$$

2.14. Measuring the activity of CAT

CAT enzyme activity was measured using H_2O_2 (65 mmol·L^{-1}) as a substrate. The blank groups contained CAT buffer; the cell lysate was added to the experimental control groups after the water bath of 37°C, while the experimental groups were added to the cell lysate before the water bath. One unit of CAT enzyme activity was defined as 1 μmol H_2O_2 catalyzed in 1 min at the temperature of 37°C. The CAT enzyme activity was calculated according to equation (S3).

$$\text{CAT activity }(\text{U}\cdot\text{mg}^{-1})=\frac{\left(OD_{\text{Experimental control group}}-OD_{\text{Experimental group}}\right)\times 650}{OD_{\text{Blank group}}\times\text{The protein content of cell lysate }(\text{mg}\cdot\text{mL}^{-1})} \quad \text{(S3)}$$

2.15. Measuring the activity of GSH-Px

GSH-Px enzyme activity was measured using H_2O_2 as a substrate. GSH working solution, cell lysate and oxidation working solution were added to the experimental groups, while the experimental control groups contained no cell lysate. Double-distilled water (ddH_2O) and acidic precipitant were added to the blank groups. The supernatant was collected by centrifugation at 3500 rpm for 10 min. The groups were added GSH-Px buffer and benzoic acid color solution sequentially and placed at room temperature for 1 min. The absorbance values were measured at 422 nm, and the GSH-Px enzyme activity was calculated according to equation (S4).

$$\text{GSH-Px activity } (\text{mU}\cdot\text{mg}^{-1}) = \frac{(OD_{\text{Experimental control group}} - OD_{\text{Experimental group}}) \times 200}{(OD_{\text{Experimental control group}} - OD_{\text{Blank group}}) \times \text{The protein content of cell lysate } (\text{mg}\cdot\text{mL}^{-1})} \quad (S4)$$

2.16. Measuring the activity of LDH

The LDH enzyme activity was determined based on the color reaction of pyruvate with dinitrophenyl hydrazine. The blank groups contained distilled water, and the standards groups contained different concentrations of pyruvate standards. Distilled water and cell lysate were added to the experimental control groups, and the experimental group contained cell lysate and nicotinamide adenine dinucleotide buffer. After the plates were placed at room temperature for 5 min, the absorbance values were measured at 450 nm. The LDH standard curve was constructed using the pyruvate standard as the horizontal coordinate and the OD Standard group-OD Blank group as the vertical coordinates. The LDH enzyme activity was calculated from the standard curve's OD $_{\text{Experimental group}}$-OD $_{\text{Experimental control group}}$.

2.17. Measuring the content of MDA

The MDA content was determined by the reaction based on thiobarbituric acid (TBA). In brief, RIPA lysate was used as the blank groups, different concentrations of MDA standards as the standards groups and experimental groups for cell lysate. The samples of the different groups and the MDA assay working solution (containing TBA) were mixed and heated at 95°C for 40 min. The absorbance values were measured at 535 nm after cooling and centrifugation at 4000 rpm for 10 min. The MDA standard curve was constructed using the MDA standard as the horizontal coordinate and the OD $_{\text{Standard group}}$-OD $_{\text{Blank group}}$ as the vertical coordinate. The MDA content was calculated from the OD $_{\text{Experimental group}}$-OD $_{\text{Blank group}}$ in the standard curve.

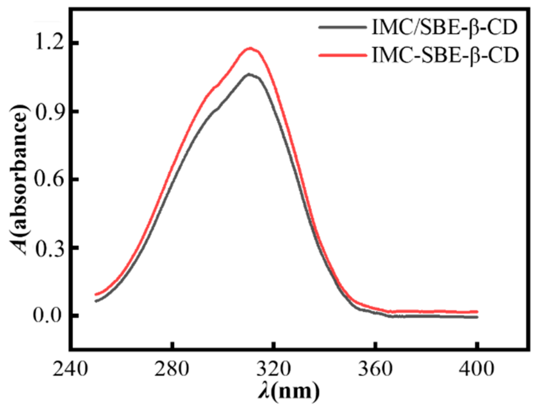

Figure S1. UV-vis spectra of IMC- SBE-β-CD and IMC/SBE-β-CD.

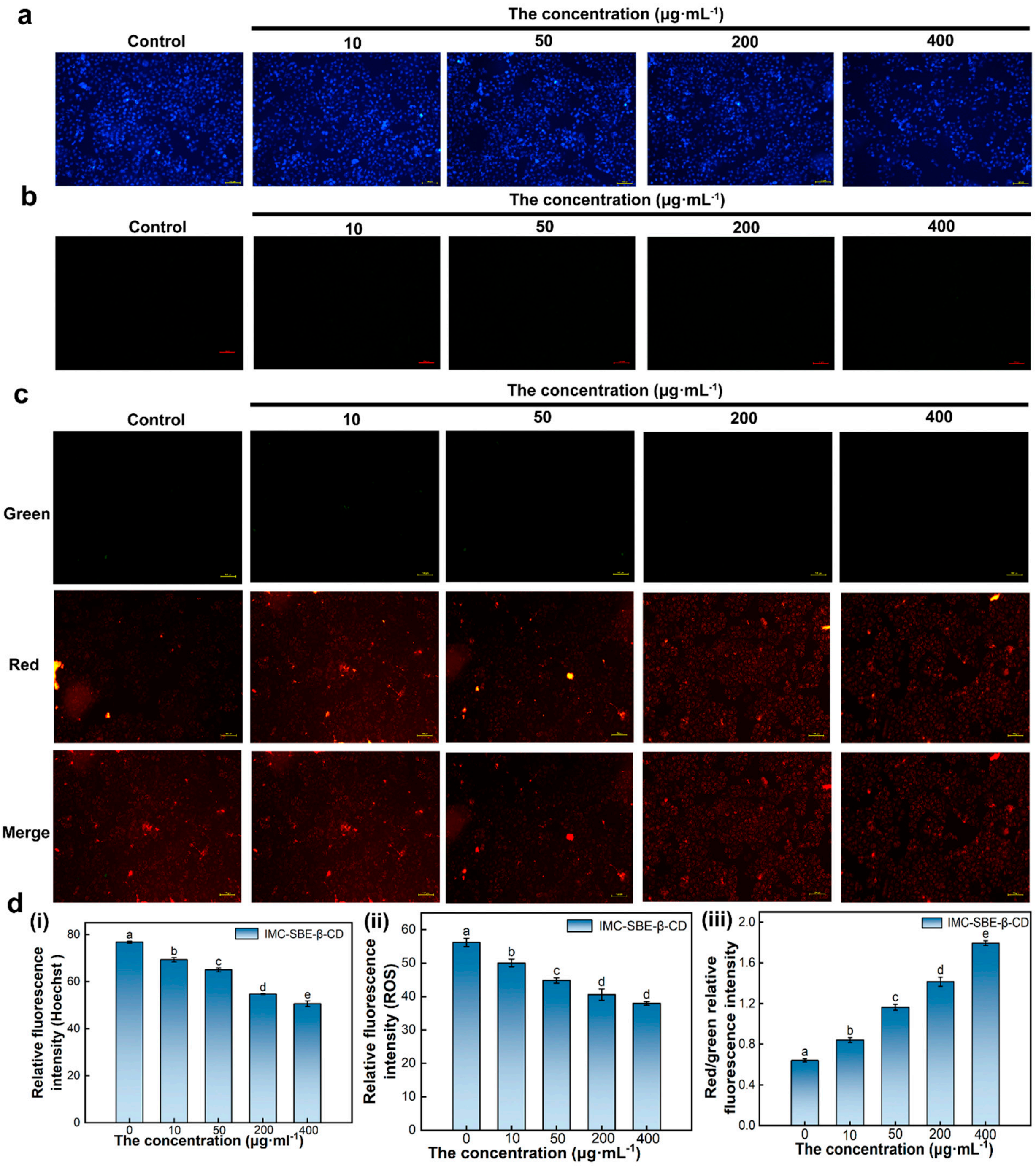

Figure S2. Effect of IMC-SBE-β-CD on nuclear fluorescence, ROS and MMP of the cells. a: The effect of IMC-SBE-β-CD on apoptosis (Scale bar: 100 μm), b: The effect of IMC-SBE-β-CD on ROS level (Scale bar: 100 μm), c: The effect of IMC-SBE-β-CD on MMP (Scale bar: 100 μm), d: Relative flu-orescence intensity (Lowercase letters denote $p<0.05$). ((i) Hoechst, (ii) ROS, (iii) MMP).

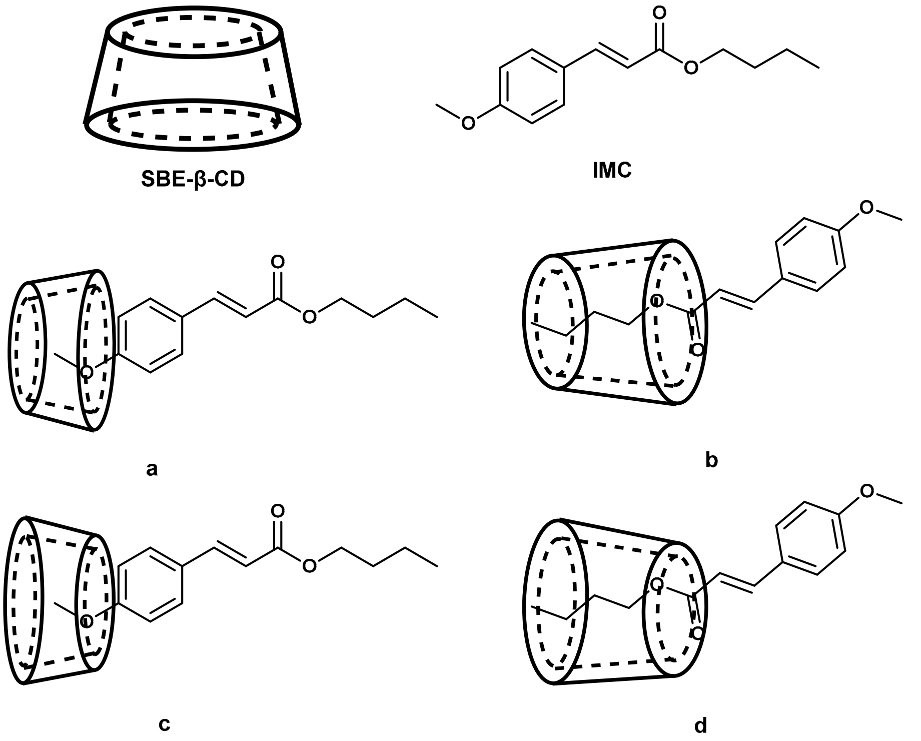

Figure S3. Schematic representation of the IMC-SBE-β-CD conformation.

Table S1. Energy values of the optimized complexes

	Complexes	$\Delta_r G$ /kJ·mol^{-1}	$\Delta_r H$ /kJ·mol^{-1}
Inclusion complexes	Inclusion a	409.4	476.3
	Inclusion b	410.7	491.6
	Inclusion c	407.2	484.2
	Inclusion d	422.8	498.9
IMC outside	Exo1	358.5	416.0
	Exo2	358.2	425.3

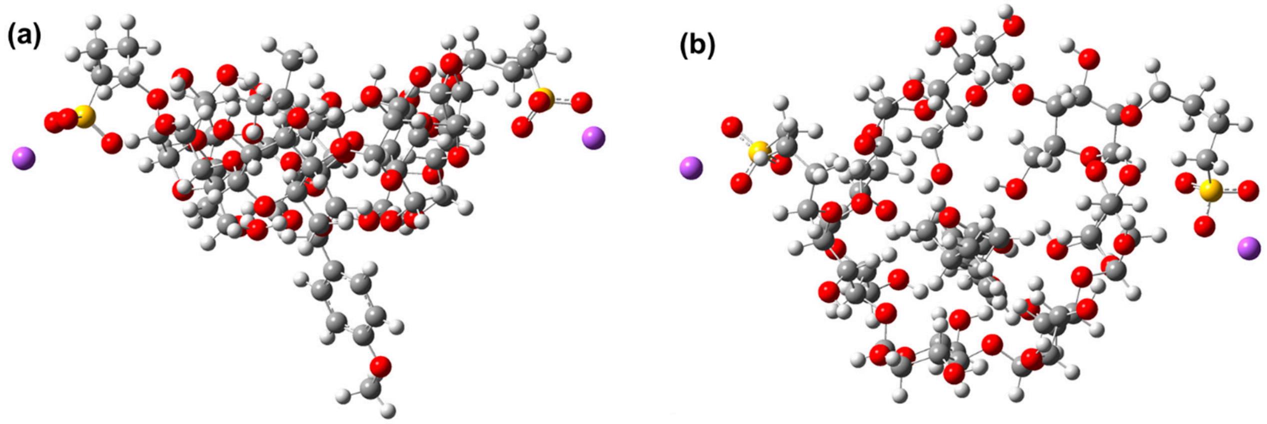

Figure S4. The lowest conformer of the complex (a) side view; (b) top view.

Table S2. Forward and reverse sequence of different primers.

Gene name	Sequences (5'-3')	Product size
β-actin-F	CACATTGGCAATGAGCGGTTC	145 bp
β-actin-R	AGGTCTTTGCGGATGTCACGT	
Bcl-2-F	ATCGCCTGTGGATGACTGAGT	135 bp
Bcl-2-R	GCCAGAGAATCAAACAGAGC	
Bax-F	CCCGAGAGGTCTTTTCCGAG	116 bp
Bax-R	CCCGCCCATGATGTTTTGAT	
Caspase-3-F	TGGACTGTGGCATTGAG	103 bp
Caspase-3-R	CCAGGTGTGGAGTA	

Cartesian coordinates for stationary points

Optimized at PM6, cartesian coordinates in Å.

Inclusion_a

C -5.15819000 -1.09678500 -1.38105300
C -5.23039100 -2.49040700 -0.71387300
C -3.92214300 -3.29494400 -0.80722800
C -3.24010300 -3.09873900 -2.18327700
C -3.07629300 -1.60172700 -2.50466400
C -2.46380500 -1.27162200 -3.86969700
O -5.48305000 -2.21538800 0.67814300
O -4.31058100 -4.66917600 -0.68865700
O -1.94787200 -3.70719300 -1.98278500
O -4.44778800 -1.11209500 -2.61118800
O -1.57725200 -0.15782400 -3.73473600
C -4.92237500 3.79607000 0.47681600
C -5.50082000 2.71499900 1.42439200
C -4.79462900 1.37776300 1.14790800
C -4.96244200 1.07392100 -0.35810800
C -4.18761500 2.13565700 -1.16006200
C -4.26828200 2.00626200 -2.68267300
O -5.24089900 3.20128300 2.75029600
O -5.40928300 0.36604200 1.94505400
O -4.40578200 -0.24391000 -0.50233900
O -4.88784200 3.39626600 -0.88513100
O -2.97731900 2.24339900 -3.25516300
C -0.75199400 7.03097200 1.05544200
C -1.71257300 6.87671000 2.25992300
C -2.40798200 5.50403900 2.21991300
C -3.05138900 5.30793000 0.82554100
C -1.98633700 5.40655100 -0.28478300
C -2.54492000 5.39479600 -1.71377600
O -0.89098600 7.02742400 3.42467500
O -3.42735400 5.56750400 3.21866900
O -3.55609800 3.96397900 0.88406700
O -1.40062800 6.74409100 -0.17458800
O -1.71126100 4.57548800 -2.53295100
C 4.33109700 5.59984300 0.53675000
C 3.92224400 6.30813800 1.85122100
C 2.42830900 6.07089000 2.13835400
C 1.60366800 6.46997600 0.89137600
C 2.06400800 5.69605600 -0.36107200
C 1.43163800 6.19230200 -1.66972100
O 4.75645100 5.73809100 2.86642200
O 2.11527300 6.94614000 3.22380400
O 0.26861000 6.04584100 1.22813500
O 3.48334100 5.99257600 -0.53931100
O 1.10411200 5.07509000 -2.49686300
C 5.96692700 0.63942400 -0.41909800
C 6.38366900 1.23667200 0.94475500
C 5.49158500 2.40525700 1.40719400
C 5.15042800 3.33644900 0.21949100
C 4.56740000 2.54377900 -0.96341900
C 4.27931700 3.37574700 -2.21751200
O 6.23683900 0.15148800 1.87257300
O 6.29705400 3.14425900 2.33542700
O 4.12658900 4.20612000 0.74990800
O 5.62973400 1.63737900 -1.37562900
O 2.99272200 2.96154100 -2.69785600
C 3.59745700 -3.83836600 -1.82107700
C 4.27249700 -3.81780200 -0.42616300
C 4.21940000 -2.40027400 0.17666500
C 4.81559500 -1.42577300 -0.86277100
C 3.93563500 -1.42665400 -2.12636100
C 4.45740100 -0.55646400 -3.27576300
O 3.53127500 -4.77097100 0.34614500
O 5.02477500 -2.43951400 1.35621200
O 4.77895200 -0.14062900 -0.21313500
O 4.02110200 -2.78340600 -2.66995100
O 3.40018200 0.25208700 -3.81410400
C -1.51534000 -4.61878000 -2.99176400
C -0.79201000 -5.76803500 -2.23746500
C 0.47936600 -5.26064500 -1.53461100
C 1.30095800 -4.32658300 -2.45698300
C 0.43514200 -3.22421000 -3.09108900
C 1.15587800 -2.33968700 -4.10709200
O -1.77495900 -6.27877300 -1.33842100
O 1.26874900 -6.42332100 -1.26630000
O 2.20676800 -3.65791300 -1.55285500
O -0.61999900 -3.89494800 -3.83777700
O 1.05425300 -1.01424600 -3.54793500
C -6.49880200 -3.04525500 1.29635900
C -7.89714200 -2.71307000 0.77593000
C -8.74646900 -1.87107300 1.73631800
C 7.27477000 0.10250500 2.90113400
C 7.15375400 -1.30853800 3.46680800
C 8.27196300 -2.26097000 3.01701800
H -3.49734500 -5.25037100 -0.79209400
H -0.65019600 -0.46507300 -3.48230000

H -5.27138800 2.44438100 3.38239200
H -5.24462000 -0.53551300 1.49475700
H -2.42164600 1.40020200 -3.22112700
H -1.38027700 6.68069500 4.21238300
H -4.00089700 4.74079000 3.15448800
H -2.13663400 3.65402500 -2.65917400
H 4.38538600 5.96701200 3.75515800
H 1.12816800 6.90438100 3.40760000
H 0.12504800 4.82229300 -2.38601700
H 2.36165000 3.74629100 -2.71880000
H 3.64627300 -4.57662400 1.30685400
H 5.29773700 -1.48434800 1.59815100
H 3.30653700 1.10305100 -3.29411700
H -1.38838400 -6.43359400 -0.42337800
H 1.99192800 -6.18106700 -0.62505500
H 1.82486400 -0.44242600 -3.88225100
C -8.35141800 -0.39730100 1.78791400
C 8.07277900 -2.83148300 1.61317900
O -10.20656100 0.31388000 0.00130900
O -7.88085600 -0.01589600 -0.79347600
O 9.60623800 -0.72711900 0.87573700
O 9.95374700 -2.83609100 -0.24076600
O -8.50232700 1.93874000 0.55619700
O 7.88472100 -1.55638300 -0.68142000
S 8.88351200 -1.93921500 0.34515900
S -8.73674900 0.46219500 0.31626200
Na 11.36718000 -1.20513900 -0.83457500
Na -10.46588400 2.37034500 -0.89492100
H 6.96739400 -2.89370000 1.39706600
H 8.37807200 -3.89950900 1.60253700
H 9.25355200 -1.74956400 3.11158300
H 8.31609400 -3.10786700 3.73502300
H 7.13248000 -1.26253500 4.57296300
H 6.15721900 -1.72406000 3.17582900
H 7.02940800 0.89019000 3.63497500
H 8.26023300 0.31187400 2.43979700
H -7.24896800 -0.31073400 2.00086000
H -8.80577800 0.08001700 2.67990000
H -9.81790400 -1.96723800 1.45199600
H -8.68046700 -2.29522000 2.76018200
H -7.80712500 -2.18335800 -0.20422900
H -8.43859200 -3.65055700 0.54227500
H -6.22771700 -4.10455100 1.12898300
H -6.35157700 -2.79022600 2.36203700
H -6.04494000 1.05671200 -0.66155500
H -3.71059400 1.41488000 1.44263200
H -6.61817500 2.63119300 1.31150500
H -3.12685000 2.24191900 -0.82422100
H -4.62972100 1.00772600 -3.00410500
H -4.90554600 2.80260700 -3.11752700
H -5.49667800 4.74292800 0.46398600
H -3.58788300 5.02190000 -1.75152400
H -2.47801700 6.40563600 -2.16732500
H -1.17907000 4.64777800 -0.15333200
H -3.87782500 6.03490800 0.66289000
H -1.71152700 4.66816900 2.46498400
H -2.46446700 7.70232700 2.27515600
H -0.35637000 8.05788100 0.92092200
H 0.52226000 6.79912200 -1.49146500
H 2.17106300 6.76709000 -2.26620400
H 1.94455700 4.59419400 -0.23693100
H 1.64228000 7.57018900 0.72951800
H 2.21748600 5.01912000 2.44347900
H 4.14959300 7.40012500 1.80314300
H 5.35030200 5.85267600 0.18197700
H 4.27578600 4.46374900 -2.01793700
H 4.99928800 3.13363300 -3.02593700
H 3.67338800 1.94446300 -0.66808300
H 6.04685400 3.92089500 -0.08605400
H 4.57496000 2.04250100 1.92456700
H 7.45079300 1.56927800 0.89806900
H 6.78838400 0.05354800 -0.91591000
H 4.76927400 -1.18751200 -4.13414900
H 5.29074400 0.10451500 -2.96592900
H 2.86327000 -1.20579000 -1.90480200
H 5.88388200 -1.69940800 -1.10495100
H 3.18797600 -2.10657500 0.48193200
H 5.32481500 -4.18441400 -0.48892100
H 3.80971400 -4.74934500 -2.41769100
H 0.64489500 -2.34353100 -5.08873300
H 2.22522100 -2.60033000 -4.22488000
H -0.04319500 -2.58298000 -2.30349300
H 1.85171300 -4.91789800 -3.21988100
H 0.23634800 -4.74871400 -0.56369500
H -0.54587400 -6.60458700 -2.93658300
H -2.32294600 -4.95557300 -3.66856900
H -3.24207200 -0.90954000 -4.57373300
H -1.92140700 -2.13171000 -4.30800600
H -2.55408500 -1.04860700 -1.68666800
H -3.80428600 -3.62432800 -2.98473600
H -3.23004100 -3.03938200 0.02759900
H -6.06416700 -3.07871800 -1.16572400

H -6.16556400 -0.67622900 -1.64176400
H 5.77765300 3.93185300 2.66511600
O -2.76983300 -5.61442300 3.18739900
O -1.42796800 -6.18673600 1.46236800
O -0.62744800 1.88771000 -0.77365300
C -1.04215500 -8.26363200 4.68845500
C -2.25011100 -7.31004500 4.81836600
C -2.98675700 -7.02912200 3.51474100
C -1.46669000 -9.63259600 4.14212700
C 0.06928800 -7.65297700 3.83097200
C -1.94329800 -5.31008500 2.14110200
C -0.71905700 -1.94738500 0.81227300
C -1.82590600 -3.85916900 1.98551500
C -0.87639900 -3.34526400 1.18250300
C -1.62687200 -0.94550600 1.21835700
C 0.33110100 -1.60492400 -0.06394600
C -0.53973200 0.62273400 -0.25272700
C -1.52996100 0.34201100 0.71106000
C 0.41864600 -0.33460200 -0.62059500
C 0.19913600 2.18000500 -1.93078400
H -0.63758300 -8.41556100 5.72292400
H -1.90685000 -6.34228100 5.24488500
H -2.95921600 -7.72099100 5.56223500
H -2.65615000 -7.66221200 2.67012200
H -4.08405500 -7.07249900 3.61850000
H -0.63191600 -10.34147100 4.16369900
H -1.79941800 -9.56756600 3.09915300
H -2.28467000 -10.06606600 4.72464200
H -0.25252800 -7.50893300 2.78694800
H 0.95509800 -8.29415700 3.80574500
H 0.37573400 -6.67143400 4.20432900
H -2.55347700 -3.25948000 2.53431300
H -0.16010700 -4.03699600 0.68675200
H -2.42884000 -1.18707200 1.91962800
H 1.08507000 -2.35678100 -0.33372000
H -2.22792400 1.12780900 1.01741800
H 1.20504400 -0.11494300 -1.34386100
H -0.09713800 3.22043000 -2.16189600
H -0.05521100 1.50940700 -2.75978200
H 1.26372800 2.12497400 -1.67038100

Inclusion_b

C -4.93942100 -3.11550400 -0.73130900
C -4.91040200 -4.06979700 0.48901100
C -3.48029900 -4.43601700 0.93804600
C -2.59097700 -4.72757600 -0.29714200
C -2.64940700 -3.56385100 -1.29981800
C -1.81523100 -3.74198000 -2.56646900
O -5.52958700 -3.31884300 1.55045800
O -3.62556800 -5.65691800 1.67641300
O -1.24452200 -4.81804300 0.22184800
O -4.03440500 -3.52955400 -1.75258600
O -1.00642600 -2.55104000 -2.61770800
C -5.40536600 2.11844000 -1.18106300
C -6.07733800 1.45565500 0.04617700
C -5.29257600 0.20140400 0.46107100
C -5.14841200 -0.70668600 -0.77895500
C -4.30941800 0.03014000 -1.83732500
C -4.11766200 -0.72470800 -3.15334400
O -6.04814500 2.45612900 1.07897200
O -6.04166200 -0.45898700 1.48567700
O -4.45487900 -1.85506700 -0.25556900
O -5.11064500 1.20038200 -2.21951000
O -2.76897200 -0.59324400 -3.61708600
C -1.52009000 5.72386900 -1.69390700
C -2.59699300 6.00845300 -0.61712800
C -3.24727500 4.70491900 -0.12518400
C -3.68897400 3.84243200 -1.33158500
C -2.48247200 3.54622900 -2.24394400
C -2.81593800 2.83716400 -3.56263300
O -1.90730800 6.68922200 0.43865800
O -4.40652300 5.11323500 0.60987500
O -4.15679600 2.63896100 -0.69746700
O -2.00681500 4.85398900 -2.70162100
O -1.79822800 1.88431400 -3.85456100
C 3.72772100 5.03301400 -1.43849400
C 3.10095800 6.05579400 -0.45746600
C 1.63287800 5.70575300 -0.14463000
C 0.87526500 5.46423200 -1.47265100
C 1.55599700 4.33301600 -2.26448200
C 0.95373600 3.99855400 -3.63241000
O 3.91854800 6.00580500 0.71739800
O 1.11036500 6.86322000 0.51187600
O -0.43863300 5.05360800 -1.04201300
O 2.89362800 4.83287300 -2.57537900
O 0.85953400 2.57696400 -3.72395500
C 6.34833600 0.50318800 -0.56782200
C 6.63032700 1.65787000 0.42072900
C 5.48941900 2.69119700 0.45930800
C 5.01657600 3.03086800 -0.97493300
C 4.68180500 1.76929700 -1.79056300
C 4.36715200 2.03415600 -3.26785900

O 6.71840400 1.02291300 1.70502500
O 6.07077600 3.87335600 1.02586300
O 3.80173800 3.78455200 -0.75395000
O 5.90338100 0.97784300 -1.82996800
O 3.38810700 1.10618300 -3.73498200
C 4.46292100 -4.36048000 0.21939400
C 5.22331600 -3.70589000 1.39944800
C 5.05384700 -2.17615200 1.35417900
C 5.49519900 -1.69143800 -0.04554500
C 4.59419700 -2.33421600 -1.11498000
C 4.94786000 -1.99587400 -2.56555200
O 4.67188700 -4.28466400 2.59027900
O 5.90630500 -1.65510000 2.37498200
O 5.27009500 -0.27042000 -0.01538700
O 4.80896800 -3.78042400 -1.02700400
O 3.74334000 -1.65422800 -3.25681800
C -0.56393400 -6.03270300 -0.08728700
C 0.25927000 -6.40451000 1.17014900
C 1.36345400 -5.36133000 1.42624600
C 2.17396700 -5.15800800 0.12382700
C 1.25708300 -4.71696800 -1.03357200
C 1.95638200 -4.61222200 -2.39598600
O -0.69139700 -6.45224500 2.23992200
O 2.19778500 -5.94479500 2.42896100
O 3.07260500 -4.07686200 0.43524800
O 0.28026200 -5.78657100 -1.20964500
O 1.56745800 -3.39953300 -3.04202800
C -6.63434000 -3.97515500 2.21916600
C -7.86478300 -4.08649000 1.31860900
C -9.00884900 -3.13574400 1.69449400
C 7.76486800 1.56068600 2.57176900
C 7.87712900 0.51958600 3.68091100
C 9.12179200 -0.37471400 3.57761700
H -2.73668700 -5.92065700 2.04628400
H -0.09539900 -2.76798600 -2.99717600
H -6.27581500 2.02335700 1.94007900
H -5.67991800 -1.40653700 1.57260100
H -2.15121800 -1.21166900 -3.11041800
H -2.46671100 6.68133500 1.25359100
H -4.94095800 4.29026800 0.85868500
H -2.07532800 0.95651600 -3.52651800
H 3.45324000 6.46890400 1.45669900
H 0.11687600 6.76056000 0.61971300
H -0.10223200 2.27792300 -3.59334600
H 2.45004400 1.44797600 -3.54476100
H 4.76429700 -3.64519000 3.33850500
H 6.07043800 -0.66311100 2.18868300
H 3.64312200 -0.64527000 -3.31700600
H -0.21116400 -6.42564100 3.10373900
H 2.99702300 -5.35278500 2.58029200
H 2.26210500 -2.66706700 -2.90315100
C -8.79803200 -1.69563800 1.23242900
C 8.98992800 -1.50716100 2.55980600
O -10.27359800 -1.98352900 -0.97449000
O -7.81968500 -2.26221900 -1.16643500
O 10.16612400 0.27406300 0.89705400
O 10.81172500 -2.04334900 0.72314700
O -8.83135700 -0.04597000 -0.84104500
O 8.56323800 -1.35136100 -0.03370000
S 9.62830600 -1.13226700 0.97372600
S -8.91593600 -1.51776900 -0.50210200
Na 11.90231600 -0.64570100 -0.65144500
Na -10.40740600 -0.53529000 -2.70404300
H 7.90606400 -1.80652800 2.47912800
H 9.46148500 -2.42652600 2.96782100
H 10.01317600 0.25499600 3.36929100
H 9.31086000 -0.82308100 4.57650600
H 7.86921200 1.02683100 4.66515200
H 6.95266000 -0.10939700 3.67210200
H 7.40816600 2.54290700 2.92917300
H 8.69659700 1.68569400 1.98558200
H -7.79229100 -1.33009900 1.58270000
H -9.49959100 -1.02472100 1.76798500
H -9.96153200 -3.53183300 1.27797300
H -9.15198100 -3.14146000 2.79529000
H -7.56383900 -3.90350500 0.25720800
H -8.24201400 -5.12728300 1.32882300
H -6.28682600 -4.95945500 2.58134200
H -6.78015600 -3.29674000 3.08088700
H -6.14298900 -1.02195600 -1.19177600
H -4.29507300 0.45484900 0.89160800
H -7.15536600 1.21323200 -0.16749800
H -3.33242600 0.39636700 -1.42575800
H -4.36031100 -1.80312800 -3.05792800
H -4.71880400 -0.26954100 -3.96652600
H -6.02795600 2.88909600 -1.67749100
H -3.80324600 2.33405100 -3.52839500
H -2.77626600 3.55244800 -4.41070700
H -1.65917100 3.02859200 -1.69749500
H -4.51181200 4.33421500 -1.89610800
H -2.57087900 4.13235000 0.55397200
H -3.37270200 6.70756900 -1.01445900

H -1.19895700 6.62847500 -2.25124700
H -0.04262100 4.45638800 -3.78252500
H 1.64359000 4.29479000 -4.44910100
H 1.65942600 3.39304500 -1.64737800
H 0.81550000 6.39904000 -2.07402600
H 1.54247000 4.83025900 0.53956800
H 3.17308800 7.09051800 -0.87289800
H 4.69660700 5.35970000 -1.86731400
H 4.00527300 3.06814100 -3.43694800
H 5.25333800 1.82287300 -3.90153400
H 3.87876300 1.14894500 -1.30173700
H 5.77792900 3.65577400 -1.49353800
H 4.64553000 2.35012600 1.10034200
H 7.59582200 2.15511000 0.16055800
H 7.25185700 -0.11948600 -0.80765800
H 5.33385000 -2.89088300 -3.09539500
H 5.67274700 -1.16205200 -2.64232100
H 3.50591500 -2.13973100 -0.91170200
H 6.58639600 -1.90837300 -0.22699600
H 4.01152800 -1.85851700 1.59176700
H 6.30356800 -3.98991400 1.37513800
H 4.67205000 -5.44092100 0.08730400
H 1.60040400 -5.41271800 -3.07783400
H 3.06011900 -4.64609600 -2.30581900
H 0.70051100 -3.77814700 -0.79036100
H 2.73970600 -6.08083400 -0.13631800
H 0.95602400 -4.39617400 1.80837700
H 0.69978300 -7.42579500 1.06762500
H -1.23967900 -6.83843100 -0.44119200
H -2.45022100 -3.75922900 -3.47375100
H -1.16308800 -4.63546000 -2.53658100
H -2.40533000 -2.58055800 -0.81547500
H -2.89395700 -5.68713300 -0.77368600
H -3.03798400 -3.65283100 1.59385500
H -5.47919100 -5.00066200 0.26273800
H -5.93486200 -3.04782100 -1.24055800
H 5.36902600 4.58125000 1.09379600
O -0.65924200 -0.99870300 1.31187900
O -1.79810200 0.32234200 -0.12843600
O -4.51313200 4.52048900 6.80554900
C 1.71332100 0.04037500 -0.80796000
C 1.45834500 -1.14024200 0.15368900
C 0.02415000 -1.64639800 0.18924000
C 1.13448300 -0.23649500 -2.19676000
C 1.18918800 1.36764700 -0.25265500
C -1.51136600 0.03298900 1.01904000
C -3.19082800 2.49888200 3.41893500
C -1.96207000 0.67002300 2.26558800
C -2.71612900 1.78115900 2.23672400
C -2.64347800 2.27250800 4.70118100
C -4.21957100 3.44541500 3.27713300
C -4.14789900 3.90813200 5.63105300
C -3.10994900 2.96756700 5.80756100
C -4.71128100 4.15503200 4.37178800
C -5.56095300 5.51978700 6.75571500
H 2.83838100 0.14295900 -0.90654000
H 1.76351300 -0.85145100 1.18064300
H 2.13661900 -1.98400300 -0.12968500
H -0.53617000 -1.45036300 -0.75449900
H -0.05608600 -2.72152000 0.45097600
H 1.30956400 0.60885700 -2.88531700
H 0.04393900 -0.38938400 -2.15894000
H 1.57905600 -1.13438000 -2.65477300
H 0.10436000 1.46443000 -0.39499400
H 1.65662400 2.22628200 -0.76765000
H 1.39646500 1.47634500 0.81516900
H -1.63155000 0.18129200 3.18340600
H -3.03307000 2.20424300 1.26272400
H -1.83861600 1.54451400 4.82317800
H -4.65243000 3.63165800 2.28132200
H -2.69718200 2.80645600 6.80098400
H -5.50574300 4.88023900 4.23240600
H -5.62627100 5.82608700 7.80815500
H -6.49957900 5.07019400 6.41973400
H -5.25751700 6.35835200 6.12233500

Inclusion_c

C 5.42356200 -2.03313500 0.54435700
C 5.45412800 -3.32667200 -0.30375300
C 4.14129500 -4.12798400 -0.26543400
C 3.55247400 -4.14527800 1.16683700
C 3.42316700 -2.71846100 1.73237800
C 2.93143600 -2.64744600 3.18315600
O 5.66877200 -2.88086300 -1.65846600
O 4.51706500 -5.46751300 -0.61428600
O 2.23520100 -4.68842400 0.95377400
O 4.79085800 -2.21737100 1.80063900
O 2.08159400 -1.51200200 3.34411800
C 5.12261800 3.06074300 -0.69429900
C 5.71498100 2.10342200 -1.75866600
C 5.03135300 0.73238400 -1.63604800
C 5.20602200 0.25362600 -0.17787100

C 4.44848700 1.21169100 0.75803700
C 4.56361000 0.90062700 2.25365100
O 5.44267900 2.73101700 -3.02077800
O 5.65571000 -0.17238000 -2.54957200
O 4.63761200 -1.07079900 -0.17773100
O 5.12161200 2.50664600 0.61304000
O 3.27787300 1.01863400 2.86886800
C 0.97959600 6.38048800 -0.90844800
C 1.96847900 6.36736200 -2.10205200
C 2.66654700 4.99919800 -2.23827100
C 3.23572100 4.58063000 -0.86090700
C 2.09940700 4.52427800 0.17771000
C 2.53179200 4.16447700 1.60002100
O 1.16689200 6.67060100 -3.25051200
O 3.73614000 5.21999800 -3.16072200
O 3.74920500 3.25313800 -1.06992700
O 1.59161200 5.89238000 0.27965800
O 1.65439000 3.09447500 1.97620600
C -4.14552000 5.00279800 -0.60469300
C -3.72270000 5.89635500 -1.79864100
C -2.23044100 5.69499100 -2.12347300
C -1.39332100 5.85789400 -0.83152600
C -1.88056400 4.88169300 0.25851900
C -1.21053200 5.04894500 1.62458500
O -4.55978400 5.49273800 -2.88884400
O -1.90336200 6.74066000 -3.04308800
O -0.06744800 5.47315600 -1.25105500
O -3.28176000 5.21702900 0.50802500
O -1.01805700 3.71726600 2.10906300
C -5.73107100 -0.07313600 -0.44520500
C -6.13073200 0.70616700 -1.71979000
C -5.26827700 1.95900100 -1.96240200
C -4.99991100 2.72124900 -0.64200600
C -4.46643500 1.78666100 0.45963300
C -4.31441500 2.43489000 1.84095000
O -5.90192700 -0.21796500 -2.79314200
O -6.06287500 2.80378700 -2.80720500
O -3.96173000 3.65329400 -1.02068800
O -5.51317900 0.79319600 0.66128600
O -3.14394000 1.90390200 2.46619700
C -3.14384100 -4.55778200 0.35547500
C -3.54581600 -4.30768300 -1.12628000
C -3.69737100 -2.80779000 -1.44609700
C -4.42898400 -2.09788000 -0.28361000
C -3.61665200 -2.25684700 1.01185100
C -4.21665100 -1.59079100 2.25326500
O -2.54019500 -4.93322200 -1.92520500
O -4.49959700 -2.73227400 -2.62572400
O -4.47597900 -0.72104700 -0.70359200
O -3.68601700 -3.68713200 1.31957800
O -3.17648600 -0.98108500 3.02536400
C 1.81586100 -5.74858100 1.81103700
C 1.07364500 -6.74686800 0.88648000
C -0.04161500 -5.99498600 0.13698400
C -0.94757600 -5.25610100 1.15179600
C -0.13982600 -4.36779600 2.11793800
C -0.95026400 -3.80366400 3.28962300
O 2.07098800 -7.24502900 -0.01391600
O -0.79847700 -7.00655400 -0.53505000
O -1.71928700 -4.34823600 0.33985100
O 0.89555300 -5.18502000 2.74312900
O -0.79006900 -2.38263100 3.20797500
C 6.70718800 -3.58488400 -2.38886700
C 8.09890500 -3.29948300 -1.82731600
C 8.96089200 -2.37798300 -2.69939300
C -6.90734200 -0.17379900 -3.85267900
C -6.65133800 -1.45606600 -4.63778000
C -7.69081100 -2.55968400 -4.39077700
H 3.70812000 -6.05523500 -0.57345000
H 1.12228900 -1.74229500 3.13389800
H 5.51775400 2.05949800 -3.73804100
H 5.55346200 -1.11992500 -2.17840800
H 2.78658200 0.13682200 2.83501000
H 1.68500300 6.47345300 -4.06920500
H 4.29153500 4.38389700 -3.22452100
H 2.16616000 2.38562000 2.48710400
H -4.20286600 5.87465000 -3.72860500
H -0.92229900 6.70124900 -3.24743200
H -0.13506600 3.62113900 2.61384300
H -2.36806700 2.54595900 2.35740600
H -1.70934400 -4.39056500 -1.90376500
H -4.82838800 -1.77235300 -2.73335300
H -3.00787200 -0.03965800 2.71440100
H 1.62288700 -7.71395200 -0.76193800
H -1.48570100 -6.57354400 -1.12080100
H -1.68693400 -1.91016400 3.29441600
C 8.60682700 -0.89694500 -2.58920700
C -7.47946500 -3.32754900 -3.08616100
O 10.45291100 -0.41091200 -0.72067800
O 8.12237900 -0.84013500 0.01395600
O -9.23025700 -1.51549700 -2.10126200
O -9.40326800 -3.79642800 -1.33791700

O 8.75061700 1.26996300 -1.07680300
O -7.48571100 -2.41810300 -0.61245500
S -8.40693800 -2.72506600 -1.73280100
S 8.98548700 -0.22306100 -1.01984200
Na -10.95097700 -2.38597700 -0.53524600
Na 10.72435100 1.53401200 0.40515000
H -6.37773600 -3.32579000 -2.83604500
H -7.67933600 -4.40673600 -3.25663600
H -8.71180000 -2.12579900 -4.44554400
H -7.63596600 -3.28204400 -5.23335800
H -6.60837200 -1.22555100 -5.71965100
H -5.62908100 -1.83209400 -4.38172200
H -6.71333600 0.74239900 -4.43743100
H -7.91673900 -0.12565100 -3.39896000
H 7.51394500 -0.75074800 -2.81144700
H 9.09716500 -0.33589100 -3.41083200
H 10.03153900 -2.52996800 -2.43589900
H 8.87966300 -2.68741200 -3.76244600
H 7.99966000 -2.85896900 -0.80370400
H 8.63880700 -4.25397900 -1.66992200
H 6.46081100 -4.66288300 -2.37507300
H 6.54963100 -3.18033300 -3.40564800
H 6.29274300 0.19250600 0.10812000
H 3.94863500 0.78471600 -1.93269300
H 6.83369300 2.02543400 -1.65663000
H 3.37935500 1.34926700 0.46293000
H 4.96906500 -0.11384000 2.44343700
H 5.18198600 1.66400500 2.76783300
H 5.67982400 4.01161600 -0.57890500
H 3.58578300 3.82970500 1.65596400
H 2.35648200 5.00386000 2.30059200
H 1.26012800 3.85822300 -0.14734400
H 4.04626600 5.27152800 -0.53839300
H 1.98723500 4.21793300 -2.65078100
H 2.72521600 7.18173500 -1.99400800
H 0.61680000 7.39027000 -0.63142300
H -0.23432000 5.56698600 1.55322900
H -1.87825600 5.56935200 2.33984300
H -1.82383700 3.81728300 -0.07908900
H -1.40713100 6.91106900 -0.47596900
H -2.03516700 4.70945200 -2.60765000
H -3.93869600 6.97053800 -1.58487800
H -5.15895100 5.21780300 -0.21372900
H -4.23949900 3.53769700 1.78282200
H -5.14665400 2.13468100 2.51032100
H -3.52797500 1.26683200 0.15370200
H -5.91387500 3.26103600 -0.31051500
H -4.31919400 1.70477400 -2.48716700
H -7.21057300 0.98884600 -1.66616700
H -6.52320300 -0.78565300 -0.08928700
H -4.65013500 -2.34941100 2.93675700
H -4.97810400 -0.82574100 1.99706900
H -2.53787100 -1.97641400 0.89143300
H -5.47724300 -2.49729100 -0.16018600
H -2.71668600 -2.32433500 -1.66625900
H -4.47342300 -4.86518400 -1.39946000
H -3.41956600 -5.56418700 0.73294900
H -0.52027500 -4.12124900 4.26067900
H -2.02230900 -4.06476900 3.23423000
H 0.36780000 -3.53782300 1.56115600
H -1.60496700 -5.97293000 1.68903300
H 0.38272800 -5.28511400 -0.61385300
H 0.66849900 -7.61046500 1.46301900
H 2.62128600 -6.18645800 2.42712800
H 3.77670800 -2.44464100 3.87438600
H 2.39206900 -3.56392500 3.49125900
H 2.82622200 -2.05026100 1.06583900
H 4.15088000 -4.79945600 1.83672600
H 3.39030700 -3.75402300 -1.00297700
H 6.29327700 -3.97782900 0.04004600
H 6.44660500 -1.64810700 0.80495600
H -5.55746900 3.64414400 -2.99646600
O -1.15061200 0.67242500 5.04250300
O -0.26510000 2.57870400 4.21089300
O 1.80405000 -2.55509600 -2.30488400
C -3.65018900 3.20143900 5.79671400
C -3.23753300 1.72202800 5.65741900
C -1.81600500 1.41692900 6.11545000
C -3.61791600 3.65614300 7.26264900
C -2.78579100 4.10915100 4.91820700
C -0.49136600 1.38936100 4.07794300
C 0.32771200 0.04273600 0.58191000
C -0.13417600 0.49244400 2.96831800
C -0.07711100 0.90650600 1.69657300
C -0.19445400 -1.25087900 0.40552200
C 1.29190900 0.52577800 -0.31705000
C 1.32934600 -1.60924100 -1.42388000
C 0.29632000 -2.08535600 -0.59235700
C 1.79785500 -0.29305200 -1.32477900
C 2.96514000 -2.20377300 -3.10230100
H -4.70829100 3.27765200 5.43505500
H -3.33634100 1.42148300 4.58379500

H -3.95619600 1.07878100 6.19584000
H -1.22233500 2.31807200 6.35087900
H -1.76770900 0.69141500 6.94381300
H -4.01213400 4.67176200 7.36869800
H -2.59835700 3.66432100 7.66202000
H -4.21601500 2.99817800 7.89981900
H -1.72618400 4.08248500 5.21269900
H -3.11869800 5.14748100 4.95031500
H -2.79452700 3.77448000 3.86984900
H 0.07877800 -0.54457800 3.27648300
H -0.28613300 1.95837100 1.41911800
H -0.99900500 -1.61305400 1.06711000
H 1.68147800 1.54586800 -0.20303300
H -0.08917600 -3.09508200 -0.72127700
H 2.56699300 0.09254200 -1.99758800
H 3.15755400 -3.13703800 -3.64434100
H 3.81702600 -1.95478500 -2.44627000
H 2.72468400 -1.38743800 -3.78634400

Inclusion1 d

C 5.57705800 -2.13213900 -0.20388000
C 5.68394900 -3.34734200 -1.15440000
C 4.39728500 -4.18854400 -1.18971800
C 3.77963200 -4.35404400 0.22154100
C 3.67832100 -3.02071000 0.98637300
C 3.24222400 -3.15054300 2.45173700
O 5.86368100 -2.77290000 -2.46222200
O 4.80695600 -5.48644800 -1.64505500
O 2.43899400 -4.78727300 -0.08961100
O 5.03856600 -2.50144500 1.06016800
O 2.42047500 -2.03554300 2.79737600
C 4.69346000 2.96278300 -1.05980000
C 5.09825200 2.12218400 -2.29335200
C 4.59226900 0.67513000 -2.16362800
C 5.04037300 0.14647200 -0.78435500
C 4.34980100 0.96390300 0.32116300
C 4.74541800 0.57306900 1.74969600
O 4.49824000 2.79293700 -3.41193600
O 5.21507700 -0.07408600 -3.21303600
O 4.62544000 -1.22948900 -0.79081500
O 4.90605800 2.31428800 0.18242900
O 3.58847900 0.59430200 2.58977900
C 0.68011900 6.27649600 -0.11148200
C 1.40692300 6.38115400 -1.47548600
C 1.95226100 4.99727300 -1.87354500
C 2.84188800 4.47824400 -0.71887200
C 2.00861300 4.33150500 0.56947300
C 2.81164300 3.97647500 1.82724600
O 0.42087800 6.85296500 -2.40159200
O 2.73095600 5.20926900 -3.04818800
O 3.27894600 3.18724300 -1.17511300
O 1.49297200 5.66636400 0.87933400
O 2.10162700 2.98205800 2.56989000
C -4.34711700 4.90190300 0.88233900
C -4.09886900 6.01036400 -0.17136800
C -2.70361200 5.81671000 -0.79316300
C -1.65253000 5.75803900 0.34170400
C -1.97897900 4.61903600 1.32719200
C -1.07981400 4.54474600 2.56730700
O -5.14012500 5.84821800 -1.14181500
O -2.48420100 6.96629100 -1.60932500
O -0.43197500 5.41112100 -0.34195000
O -3.30784300 4.89182200 1.86054100
O -0.66086800 3.18664100 2.71125000
C -5.88994900 -0.13922100 0.19773500
C -6.53373100 0.87801900 -0.77344500
C -5.79199300 2.22466000 -0.84138200
C -5.22674200 2.65803800 0.53039000
C -4.48205000 1.52003600 1.25100000
C -4.04665300 1.84948000 2.68406100
O -6.41832400 0.24927300 -2.06089100
O -6.78303300 3.18516500 -1.23584300
O -4.25984600 3.67224900 0.17198200
O -5.47407900 0.46850800 1.41659400
O -2.75136300 1.30085800 2.93382100
C -3.04886400 -4.49292800 -0.38868100
C -3.59318400 -3.93952600 -1.73738200
C -3.89897200 -2.42940700 -1.68979100
C -4.50693900 -2.05012100 -0.31986200
C -3.50343000 -2.40521400 0.78766000
C -3.92055300 -2.06793300 2.21981700
O -2.61608800 -4.26161000 -2.72810200
O -4.86905300 -2.18209300 -2.71218100
O -4.69979100 -0.62735600 -0.43412300
O -3.48992400 -3.87197300 0.79183600
O -2.79494100 -1.53322100 2.92638200
C 1.95789400 -5.94620600 0.58473300
C 1.15212200 -6.72924500 -0.48300400
C 0.04818000 -5.81302500 -1.04241300
C -0.80259000 -5.26194300 0.12747600
C 0.06407700 -4.55034200 1.18562200
C -0.66809700 -4.20680900 2.49069200

O 2.10766200 -7.08703800 -1.48895700
O -0.76231600 -6.65058900 -1.87383800
O -1.62947100 -4.25278300 -0.48179100
O 1.09554900 -5.49040000 1.62061300
O -0.42205000 -2.83344800 2.79359300
C 6.91362500 -3.36652200 -3.26440000
C 8.29493800 -2.91453300 -2.79143100
C 8.91186800 -1.80422000 -3.65194200
C -7.57987800 0.42588700 -2.92774200
C -7.36829600 -0.62401100 -4.01430900
C -8.30469300 -1.83651200 -3.90466200
H 3.99973400 -6.06297800 -1.75426500
H 1.44812400 -2.23723900 2.64998900
H 4.55270500 2.20912700 -4.20449900
H 5.17848600 -1.05798000 -2.96726300
H 3.17985400 -0.33191100 2.65337300
H 0.72427800 6.65780300 -3.32388500
H 3.25356300 4.37133900 -3.25152400
H 2.44679400 2.05184500 2.33168700
H -4.89378900 6.34150500 -1.96364000
H -1.55705900 6.92515800 -1.99250500
H 0.31032300 3.07230900 2.44549500
H -2.03077900 1.94947000 2.66793000
H -1.78745500 -3.73990300 -2.56199800
H -5.27289500 -1.25842200 -2.55413100
H -2.69909100 -0.54034900 2.71869300
H 1.62485800 -7.38532400 -2.29945800
H -1.45893600 -6.09343100 -2.32517900
H -1.22799700 -2.27177300 2.51778700
C 8.28234600 -0.43162300 -3.42604100
C -7.87635000 -2.85706600 -2.85061600
O 10.21675800 0.30806200 -1.73301200
O 8.08100500 -0.51855400 -0.78593900
O -9.55669500 -1.45093600 -1.26461300
O -9.49205900 -3.84852600 -1.01126800
O 8.23214000 1.68997900 -1.85112200
O -7.56568800 -2.49791800 -0.25753400
S -8.62393200 -2.63697900 -1.28524800
S 8.71493700 0.25586000 -1.87933100
Na -10.96764900 -2.77657500 0.29517400
Na 10.24879300 2.18599800 -0.47056500
H -6.75158500 -2.83391000 -2.75403700
H -8.05702700 -3.88307500 -3.23612800
H -9.34475000 -1.48702800 -3.73106400
H -8.32960100 -2.34518900 -4.89233200
H -7.48573600 -0.15654300 -5.01066300
H -6.30178500 -0.96210700 -3.97701100
H -7.54180700 1.46102700 -3.30822200
H -8.50674100 0.27531900 -2.33965400
H 7.16168200 -0.51177700 -3.50650200
H 8.53714500 0.24234900 -4.26885800
H 10.00690800 -1.75951400 -3.46023400
H 8.81868200 -2.06996000 -4.72572400
H 8.22434500 -2.56897500 -1.73101700
H 8.98433300 -3.78015800 -2.76498400
H 6.79755200 -4.46530000 -3.24542500
H 6.64682700 -2.98623700 -4.26828200
H 6.15859500 0.19593600 -0.66508600
H 3.48994700 0.59745800 -2.30461100
H 6.21620200 2.14804800 -2.43545700
H 3.24259500 1.02212000 0.19616600
H 5.21983900 -0.42718400 1.80019800
H 5.41930500 1.33590700 2.19148100
H 5.26913000 3.90702000 -0.95973800
H 3.82869700 3.60677200 1.58748600
H 2.86621300 4.84491600 2.51601700
H 1.14894200 3.63138700 0.43184500
H 3.71474400 5.15136500 -0.55803200
H 1.12150000 4.27703500 -2.10602300
H 2.22048200 7.14350500 -1.44025400
H 0.39771300 7.25047700 0.33548800
H -0.19853600 5.20894500 2.49690000
H -1.66033400 4.76515000 3.48733200
H -1.99545800 3.62371000 0.80035800
H -1.55837800 6.73904800 0.85445700
H -2.66150400 4.89008400 -1.43254500
H -4.20729800 7.02613000 0.27528200
H -5.27702200 5.02201200 1.47005200
H -4.02656300 2.93870900 2.87959500
H -4.69976600 1.33526400 3.42004700
H -3.62409500 1.12475200 0.63708300
H -6.03444100 3.08403700 1.16501800
H -4.98563400 2.18776400 -1.62010200
H -7.60471700 1.04013400 -0.50258300
H -6.58627900 -0.95840900 0.51750700
H -4.18319300 -2.98495400 2.78539900
H -4.75676100 -1.34165400 2.26361900
H -2.47327700 -2.02539200 0.57037500
H -5.50005400 -2.55638200 -0.15401600
H -2.99795600 -1.81238000 -1.91718100
H -4.49443300 -4.50526700 -2.07645800
H -3.28124800 -5.56551000 -0.22089100

H -0.23308400 -4.76946900 3.34207400
H -1.75778700 -4.38801000 2.42294600
H 0.56178300 -3.64835400 0.75578800
H -1.42280500 -6.06833400 0.57727100
H 0.47522200 -4.98521600 -1.65549200
H 0.72760600 -7.67041000 -0.06313800
H 2.74074200 -6.53111300 1.10152600
H 4.11541700 -3.06029500 3.13116100
H 2.69486400 -4.09321400 2.64403500
H 3.04168200 -2.27977000 0.44493500
H 4.32933700 -5.12445000 0.80317200
H 3.65500400 -3.76395600 -1.90486200
H 6.55107200 -3.98532500 -0.86694600
H 6.56270800 -1.65074100 0.02620300
H -6.34436500 4.07172700 -1.35999500
O 0.71108800 1.16041500 0.01743800
O -1.07637000 -0.17335200 0.44726000
O 2.42854500 -0.15912600 8.30368700
C -2.01125300 2.47099200 -2.11799200
C -0.74529800 2.61457300 -1.25217500
C 0.23493400 1.45190600 -1.33299200
C -1.68178100 2.64214700 -3.60552400
C -2.73996800 1.15361800 -1.85776900
C -0.07197900 0.37942100 0.84598100
C 0.55599400 -0.01377100 4.60543300
C 0.55978800 0.36848400 2.16363000
C -0.08831800 0.04215600 3.29123900
C 1.37194800 1.03632700 5.07152000
C 0.35676500 -1.15524700 5.39891500
C 1.76658500 -0.20656400 7.09497700
C 1.97647100 0.95206900 6.31896900
C 0.96428600 -1.26562400 6.65091900
C 2.29852200 -1.29366200 9.19240500
H -2.69702000 3.31962100 -1.81507800
H -1.06242400 2.75061500 -0.18818900
H -0.22424000 3.56637300 -1.51623500
H -0.19994000 0.53400100 -1.76371500
H 1.18473000 1.71290100 -1.83648200
H -2.58444800 2.55747800 -4.22061900
H -0.98060300 1.87849400 -3.95725200
H -1.23291900 3.61956700 -3.80804000
H -2.24190800 0.29699200 -2.32156000
H -3.77354000 1.17527000 -2.23590600
H -2.80424200 0.91634300 -0.77648900
H 1.62426600 0.66033300 2.16550100
H -1.16205700 -0.22619400 3.28100600
H 1.52507600 1.92697200 4.45118600
H -0.26284900 -1.97501700 5.01743800
H 2.60849700 1.74934100 6.70055100
H 0.81099100 -2.15639200 7.24779700
H 2.91484900 -0.98305100 10.04538000
H 2.70519000 -2.19348500 8.72194100
H 1.25444300 -1.42529000 9.49057600

Exo1

C 5.44850900 1.41197600 0.13529100
C 5.47963500 2.37100200 1.34814600
C 4.09316600 2.92008800 1.73013500
C 3.24218300 3.25110500 0.48114400
C 3.24272500 2.11329100 -0.55361700
C 2.55886200 2.44709000 -1.88498200
O 5.95731000 1.56883400 2.44348700
O 4.36930900 4.16390500 2.39969100
O 1.90341700 3.39447900 1.00322400
O 4.64397200 1.92415000 -0.91789600
O 1.81463300 1.29375900 -2.28508300
C 5.62656100 -3.81734600 -0.28630300
C 6.21282500 -3.20152500 1.00787100
C 5.44232300 -1.91934500 1.36862100
C 5.47569600 -0.99705600 0.12943200
C 4.71458100 -1.67438100 -1.02457500
C 4.70246100 -0.89510700 -2.34352400
O 6.06374300 -4.22029800 2.00835800
O 6.12454100 -1.30834200 2.46775600
O 4.81599900 0.20109400 0.57472000
O 5.49071500 -2.87744000 -1.34250600
O 3.41423200 -1.00687800 -2.95552000
C 1.69032200 -7.31088000 -1.01300800
C 2.67813700 -7.58549500 0.14720200
C 3.29186400 -6.26938400 0.65983700
C 3.87132400 -5.48159400 -0.53996900
C 2.77748400 -5.19619900 -1.58883200
C 3.29893200 -4.57357100 -2.89201300
O 1.89923300 -8.24180900 1.15484400
O 4.35079200 -6.66370900 1.53432600
O 4.29859400 -4.24309700 0.05230800
O 2.27463800 -6.49890700 -2.02253900
O 2.41306800 -3.54285800 -3.32948000
C -3.52180800 -6.30064600 -1.06852300
C -3.01933500 -7.44709100 -0.15393600
C -1.55177700 -7.23602800 0.27060100
C -0.69735400 -6.90309800 -0.97654800

C -1.26852600 -5.65910000 -1.68135200
C -0.54370700 -5.22264400 -2.95565400
O -3.90124200 -7.43069000 0.97440200
O -1.14152500 -8.49373400 0.81481300
O 0.60277600 -6.58286000 -0.44225500
O -2.60324000 -6.04898700 -2.13022400
O -0.26073200 -3.83122900 -2.75094200
C -5.85929700 -1.63822300 -0.08008100
C -6.26287600 -2.80160200 0.85472700
C -5.21955400 -3.93435600 0.89222300
C -4.67673700 -4.23406500 -0.52716400
C -4.16974000 -2.95625800 -1.22294900
C -3.73785200 -3.13692200 -2.68445900
O -6.33667600 -2.20264900 2.15790800
O -5.93513300 -5.09333300 1.34254900
O -3.56672900 -5.12397100 -0.26773800
O -5.34105400 -2.09467800 -1.32199000
O -2.62875600 -2.28500400 -2.97191500
C -3.81585200 3.14216100 0.77752400
C -4.66765200 2.53392600 1.91911700
C -4.54029000 0.99897700 1.90319300
C -4.92827500 0.50939100 0.48990500
C -3.93548600 1.08355700 -0.53810300
C -4.25636700 0.76102900 -2.00134100
O -4.16933300 3.11856200 3.13002700
O -5.45617000 0.50452000 2.88252000
O -4.79894400 -0.92250800 0.57048800
O -4.08575100 2.53841700 -0.47609900
O -3.04112200 0.48698700 -2.70396100
C 1.27726100 4.63907000 0.69273400
C 0.43916500 5.04466000 1.92655300
C -0.73998300 4.07729300 2.13453600
C -1.51407700 3.90854200 0.80467300
C -0.58067200 3.41996600 -0.31860700
C -1.20924100 3.38776300 -1.71758200
O 1.36198400 5.00919900 3.02276800
O -1.57896700 4.71272600 3.10305300
O -2.45453400 2.85784400 1.10336100
O 0.46558600 4.42821200 -0.45395900
O -0.86443400 2.15205100 -2.34138200
C 7.01800800 2.16122500 3.23309300
C 8.34282100 2.19255800 2.47124000
C 9.34060400 1.11085700 2.90592700
C -7.47083600 -2.65302400 2.96160500
C -7.56547900 -1.60264200 4.06400200
C -8.76311800 -0.65125500 3.92299500
H 3.51886300 4.52619900 2.77715400
H 0.82525900 1.47011800 -2.22295600
H 6.13126100 -3.80629500 2.90166700
H 5.83484500 -0.33578500 2.51755700
H 2.80008200 -0.27884000 -2.62827400
H 2.39089800 -8.21735500 2.01283100
H 4.87981000 -5.84786000 1.79826800
H 2.75263400 -2.63039800 -3.03498500
H -3.53861000 -8.02140100 1.67894700
H -0.17028000 -8.44353900 1.05995500
H 0.69342500 -3.62435500 -3.01728300
H -1.75701000 -2.74506800 -2.77594800
H -4.30991300 2.49069500 3.87965300
H -5.64979700 -0.47965600 2.67935100
H -2.83465700 -0.50180900 -2.67701500
H 0.85875500 5.05438100 3.87157000
H -2.42546400 4.18090200 3.20440900
H -1.65635800 1.51712900 -2.34326400
C 9.00726400 -0.28421100 2.38331600
C -8.54096800 0.48573200 2.92616300
O 10.67727900 -0.07730500 0.30687300
O 8.27489500 0.40943000 -0.06435800
O -9.72267200 -1.22328000 1.19398900
O -10.24373600 1.12523900 1.01076400
O 9.07407100 -1.88915100 0.28108500
O -7.99936200 0.32551500 0.35434300
S -9.12045200 0.15407600 1.30849000
S 9.25387400 -0.43558200 0.65986700
Na -11.35032300 -0.20642700 -0.41346200
Na 10.87901600 -1.57126600 -1.38573700
H -7.44359000 0.74375900 2.89896700
H -8.99416700 1.41856400 3.32452700
H -9.67346100 -1.23685700 3.67233800
H -8.97097900 -0.20553000 4.91943700
H -7.60829900 -2.10422500 5.05014300
H -6.61159000 -1.01852300 4.07911300
H -7.21286300 -3.65830500 3.33787900
H -8.37591900 -2.70920400 2.32496800
H 7.93973200 -0.53819100 2.63718100
H 9.57694600 -1.04451500 2.95536400
H 10.36232100 1.40597900 2.57871600
H 9.39261400 1.07579000 4.01434700
H 8.14087300 2.09147400 1.37642200
H 8.81588600 3.18681500 2.58502800
H 6.69356000 3.16665400 3.55721400
H 7.02815600 1.47936600 4.10433700

H 6.52923000 -0.75322500 -0.17823400
H 4.39994000 -2.13477800 1.70123900
H 7.31508400 -3.00039100 0.89283700
H 3.68784100 -1.98986200 -0.73043800
H 4.95088900 0.17684100 -2.20345500
H 5.39225700 -1.35528500 -3.08044800
H 6.24741400 -4.62393500 -0.72388200
H 4.31924300 -4.15672100 -2.77684000
H 3.27595900 -5.32014700 -3.71374900
H 1.92915200 -4.61219500 -1.16174700
H 4.73375200 -6.02303000 -0.98888600
H 2.55854800 -5.65538100 1.23269400
H 3.47794100 -8.29617400 -0.17272700
H 1.37010700 -8.22092100 -1.56100400
H 0.39637100 -5.77991800 -3.13039500
H -1.20484200 -5.29679600 -3.84176900
H -1.36300700 -4.79164800 -0.97952600
H -0.63974800 -7.77408600 -1.66612500
H -1.44922400 -6.44955400 1.05362600
H -3.13570700 -8.43399900 -0.66433900
H -4.47699800 -6.52318900 -1.58487100
H -3.47299800 -4.18709400 -2.91592500
H -4.53208200 -2.77719600 -3.37225100
H -3.38644200 -2.43415900 -0.62735800
H -5.45342000 -4.74340600 -1.14004100
H -4.39727100 -3.70944500 1.60824600
H -7.25815400 -3.20683900 0.54881900
H -6.71781100 -0.97355700 -0.36942400
H -4.67751600 1.64856500 -2.51866600
H -4.94483300 -0.10195200 -2.10247000
H -2.87946400 0.83229800 -0.28936800
H -5.99267100 0.78556200 0.24109300
H -3.51910900 0.65843300 2.19455100
H -5.73448400 2.85014900 1.82547700
H -4.00825300 4.22975800 0.58462900
H -0.76069600 4.17130800 -2.36445400
H -2.31930400 3.50781300 -1.69678400
H -0.10549100 2.44370900 -0.06518100
H -2.03966500 4.84957400 0.52227700
H -0.40853700 3.09100700 2.53506300
H 0.07288400 6.09503400 1.82532100
H 1.99737300 5.42134200 0.36966700
H 3.31090700 2.60225300 -2.68506700
H 1.88996100 3.32638600 -1.80544100
H 2.84563900 1.15959800 -0.13114900
H 3.59095900 4.20578800 0.02322100
H 3.55528600 2.23575800 2.42422600
H 6.17399600 3.21711400 1.13684600
H 6.45607200 1.24056000 -0.32828600
H -5.30268000 -5.86390800 1.39816200
O -3.23772100 7.05516400 -1.13208000
O -2.59110900 7.04472200 -3.30174400
O 5.18422900 7.26008500 0.27078300
C -5.64325900 4.78220700 -2.15959500
C -5.28006500 5.78485400 -1.04179200
C -4.65186800 7.08444400 -1.52333200
C -6.75039500 5.33916500 -3.06278300
C -4.41743000 4.37615200 -2.97848900
C -2.28590500 7.06141600 -2.12845600
C 1.50791500 7.16322000 -1.65811200
C -0.95294400 7.11017300 -1.50908700
C 0.16551500 7.09765100 -2.25048100
C 1.79607200 8.09411100 -0.63818800
C 2.50214600 6.26756600 -2.08215000
C 4.00431900 7.15481600 -0.42316800
C 3.03797800 8.10163900 -0.01990200
C 3.75029600 6.24091800 -1.45598200
C 6.02701400 6.08147900 0.34509800
H -6.03511200 3.86060900 -1.65070700
H -4.57320300 5.29428400 -0.32821400
H -6.18635600 6.00921300 -0.44692300
H -4.73462400 7.23767700 -2.61596400
H -5.02753700 7.97064100 -0.98412600
H -7.07893300 4.58632900 -3.78778600
H -6.40788700 6.20669100 -3.63775900
H -7.62772700 5.64568100 -2.48592000
H -3.94271600 5.23236000 -3.47762700
H -4.67333300 3.65458800 -3.76081400
H -3.65366900 3.90095800 -2.33668900
H -0.94471100 7.14794200 -0.41776000
H 0.12405900 7.02085600 -3.34520100
H 1.03334100 8.81226800 -0.33218900
H 2.29065200 5.56347200 -2.88919800
H 3.28211600 8.80955300 0.76991600
H 4.51147900 5.53699700 -1.78294800
H 6.92957000 6.48008700 0.82382200
H 6.25074200 5.68180500 -0.64586200
H 5.53407800 5.34102300 0.99636100

Exo2

C 5.27426000 -1.56833800 -1.05324900

C 5.31584900 -2.40456500 -2.35357800
C 3.93528100 -2.96349100 -2.73947000
C 3.20579600 -3.52900300 -1.49349000
C 3.16281200 -2.51176900 -0.33570300
C 2.60292600 -3.05415200 0.98393300
O 5.73097800 -1.47444800 -3.37001400
O 4.20366000 -4.06101200 -3.62346700
O 1.87067600 -3.75395900 -1.99496100
O 4.56303300 -2.23627100 -0.02172500
O 1.88480300 -2.03563100 1.68209800
C 5.11056600 3.59436300 -0.11598500
C 5.63275700 3.16736500 -1.50955300
C 4.95825700 1.85742400 -1.95250100
C 5.14894100 0.82484100 -0.82062200
C 4.43478900 1.31564500 0.45025500
C 4.60613400 0.41435700 1.67527100
O 5.31049800 4.26243900 -2.37970200
O 5.63032800 1.42000700 -3.13957800
O 4.54117500 -0.37020300 -1.34499100
O 5.13432800 2.54454100 0.83732900
O 3.35277400 0.29528300 2.35464600
C 1.06652300 6.67694100 1.40413000
C 1.92684700 7.15979900 0.21022800
C 2.55137000 5.96346500 -0.53199100
C 3.27214700 5.05675900 0.49479800
C 2.28059900 4.55825700 1.56539800
C 2.92235600 3.77412800 2.71858800
O 1.02739400 7.89954500 -0.62405400
O 3.50884500 6.53252400 -1.42754500
O 3.73144200 3.94968600 -0.29969200
O 1.76965000 5.75766800 2.22859400
O 2.11202700 2.64240600 3.03759700
C -4.08077300 5.43301700 1.76728300
C -3.70048500 6.74765600 1.03924000
C -2.27600700 6.67313800 0.45355300
C -1.29638400 6.16731700 1.54045000
C -1.76382000 4.80119700 2.07569500
C -0.91680200 4.19873400 3.19855500
O -4.68616500 6.89633000 0.01064000
O -1.95081500 8.02127600 0.10218200
O -0.04885200 5.98804600 0.83920600
O -3.06536200 5.04931100 2.69266100
O -0.63002400 2.86101200 2.76805500
C -6.18064300 0.87081400 0.03527500
C -6.73026600 2.14876300 -0.64011400
C -5.78203500 3.35380000 -0.49971900
C -5.18268100 3.43282200 0.92623600
C -4.56246500 2.09299300 1.36827900
C -4.09722000 2.05182700 2.83009400
O -6.82283400 1.80363200 -2.03023800
O -6.60649300 4.51230300 -0.69137000
O -4.13755600 4.41976500 0.76939400
O -5.65723400 1.13299900 1.33194900
O -2.94840700 1.21276900 2.96222000
C -3.80135700 -3.44296900 -1.79711800
C -4.51642400 -2.60026100 -2.89090700
C -4.65795000 -1.12049500 -2.48449700
C -5.07398900 -1.01142400 -0.99987400
C -4.03356100 -1.69617400 -0.09909100
C -4.38759600 -1.71128000 1.39140100
O -3.74859700 -2.76465200 -4.08367500
O -5.70021700 -0.58040700 -3.30060200
O -5.08216900 0.41058500 -0.76360100
O -4.09197000 -3.11693100 -0.45948100
O -3.22560700 -1.45803400 2.18754400
C 1.28583700 -5.01396300 -1.67130600
C 0.42988000 -5.42056400 -2.89776600
C -0.74734400 -4.44708600 -3.10646300
C -1.49570100 -4.22845700 -1.76920200
C -0.51987100 -3.74935300 -0.67867900
C -1.12079400 -3.58473300 0.71447100
O 1.34016800 -5.38686600 -4.00343500
O -1.60975300 -5.11052500 -4.03921800
O -2.41183600 -3.15096900 -2.03827300
O 0.46961200 -4.81380500 -0.52360600
O -0.84070200 -2.21535800 1.05858500
C 6.81808700 -1.92633000 -4.21405400
C 8.15175800 -1.91868400 -3.46554800
C 9.04844100 -0.72204400 -3.80908700
C -8.03134500 2.28575800 -2.69475600
C -8.07056500 1.47416400 -3.98590300
C -9.15196700 0.38375100 -4.00976400
H 3.33605300 -4.45636300 -3.92115600
H 0.92991600 -1.97332600 1.35658300
H 5.34360400 3.95261300 -3.31557600
H 5.40045200 0.44389600 -3.29102600
H 2.83763100 -0.50737000 2.01251300
H 1.43218800 8.01359000 -1.51899600
H 4.04792500 5.79148700 -1.84482500
H 2.49779300 1.79756100 2.62288900
H -4.40657800 7.61762000 -0.60465700
H -1.01565000 8.04899300 -0.25797900

H 0.35836200 2.66892700 2.85609500
H -2.10452100 1.71973300 2.75851300
H -2.88734000 -2.28049900 -3.99566000
H -5.97290600 0.32607300 -2.91917700
H -3.14925200 -0.46368600 2.37944600
H 0.83536800 -5.51057400 -4.84370900
H -2.40308900 -4.53615800 -4.22874000
H -1.60152600 -1.84965300 1.61915900
C 8.59184000 0.58571300 -3.16781700
C -8.76667200 -0.89027900 -3.25833400
O 10.40771900 0.44906500 -1.20908200
O 8.09837900 -0.34041900 -0.74208700
O -10.05517300 0.30795600 -1.20421400
O -10.31601000 -2.07419700 -1.47576000
O 8.62808500 2.06153400 -0.98486000
O -8.14624900 -1.17073900 -0.71281600
S -9.31356900 -0.94520600 -1.59858300
S 8.93410000 0.65637200 -1.45548100
Na -11.50152000 -1.15984400 0.19425800
Na 10.67809200 2.10802100 0.33716800
H -7.64620000 -1.02003100 -3.30499600
H -9.12752700 -1.77528000 -3.82484600
H -10.10908100 0.80282600 -3.63267300
H -9.34981800 0.11599400 -5.07006900
H -8.21441600 2.15453300 -4.84726700
H -7.06235200 1.01525900 -4.14710700
H -7.89142100 3.36759600 -2.86374800
H -8.90678600 2.11570400 -2.03762400
H 7.48639600 0.72490500 -3.34075800
H 9.02375900 1.44478400 -3.71994000
H 10.09544200 -0.95227600 -3.51233600
H 9.08605300 -0.58886800 -4.91067000
H 7.96091300 -1.93173200 -2.36549300
H 8.70175300 -2.85677400 -3.67311100
H 6.56310600 -2.92471700 -4.61347300
H 6.76802200 -1.18114700 -5.03010100
H 6.23608900 0.63255000 -0.60903600
H 3.88295700 2.00255300 -2.20836400
H 6.75356400 3.06226100 -1.49668100
H 3.36454500 1.56323300 0.27033300
H 4.98578400 -0.59752500 1.41841200
H 5.28464500 0.88219400 2.41886000
H 5.70697700 4.39947800 0.35850800
H 3.95077100 3.43887900 2.47669700
H 2.92364200 4.38331700 3.64700200
H 1.42249800 4.00463500 1.11858200
H 4.13165000 5.59059000 0.95927200
H 1.79861100 5.39193300 -1.12288800
H 2.71974800 7.86689300 0.55481400
H 0.77293400 7.48545400 2.10534700
H 0.02455300 4.75352900 3.37336000
H -1.49614400 4.11757700 4.13992000
H -1.89234800 4.05551500 1.25066400
H -1.18882200 6.91270400 2.35908300
H -2.22994600 6.03636200 -0.46008800
H -3.79193300 7.62046800 1.72987500
H -4.99847000 5.50614400 2.38325200
H -3.86193700 3.06156000 3.22114900
H -4.85741600 1.55520200 3.46885200
H -3.76165000 1.75239400 0.67245000
H -5.95003400 3.78231900 1.65150500
H -4.98516800 3.33720300 -1.27768900
H -7.73612500 2.39798400 -0.22412300
H -6.95947100 0.07983300 0.20656700
H -4.70907400 -2.73888400 1.69368500
H -5.17421800 -0.97584400 1.65060000
H -2.99637700 -1.33065300 -0.27390400
H -6.10625400 -1.43554400 -0.83768900
H -3.72795700 -0.54137000 -2.68800400
H -5.50926100 -3.03176100 -3.16229500
H -4.01869900 -4.53005500 -1.84833500
H -0.61492600 -4.23600400 1.46246100
H -2.21298500 -3.76048500 0.74256600
H 0.00474500 -2.80971100 -0.98685800
H -2.04016500 -5.14730800 -1.45968400
H -0.40914300 -3.48007200 -3.54426800
H 0.05512700 -6.46661700 -2.78743900
H 2.02238200 -5.77950900 -1.35788000
H 3.43323200 -3.31011400 1.67884100
H 1.94570200 -3.93332400 0.83293800
H 2.66748700 -1.55887200 -0.63156300
H 3.67239800 -4.48579300 -1.16999100
H 3.31598700 -2.20555700 -3.26977000
H 6.04392900 -3.24170400 -2.24798000
H 6.28745100 -1.36093600 -0.62074100
H -6.03599400 5.32919600 -0.63441700
O -0.97034900 -5.04181800 3.34886900
O -0.36812200 -5.80751500 5.38984800
O 6.68204000 -1.74911600 2.72238400
C -4.74455600 -4.91701100 3.56530000
C -3.27433500 -4.52263500 3.80986000
C -2.29831500 -5.64204600 3.47475600

C -5.02111000 -5.17947700 2.08012200
C -5.15541800 -6.12365200 4.41886800
C -0.09095900 -5.15911200 4.40534800
C 3.55379300 -4.03662400 4.19818600
C 1.13589600 -4.41678200 4.07289700
C 2.33223000 -4.77126900 4.56072900
C 3.63487200 -2.64570500 4.42436000
C 4.60922600 -4.70212900 3.56181900
C 5.72655300 -2.59723500 3.23331800
C 4.72480400 -1.92303800 3.96473300
C 5.70133600 -3.98728700 3.06091000
C 7.54988900 -2.26150800 1.67895100
H -5.37036700 -4.04220500 3.88356500
H -3.13912800 -4.21164000 4.86484400
H -3.02139100 -3.61335000 3.21775300
H -2.27366100 -6.43873800 4.23908800
H -2.45365800 -6.07226000 2.47012300
H -6.07696200 -5.40648700 1.90362900
H -4.43598200 -6.01707300 1.69050400
H -4.77054700 -4.29524500 1.46480700
H -4.67242000 -7.04736700 4.08457300
H -6.23643500 -6.28993100 4.37445000
H -4.88625500 -5.97854200 5.47111500
H 1.00348100 -3.56420400 3.39032600
H 2.45985200 -5.62955200 5.22880200
H 2.82225800 -2.13293800 4.94302500
H 4.56971200 -5.78251000 3.42672300
H 4.78961700 -0.84546800 4.11553800
H 6.50109400 -4.50673500 2.54545100
H 7.84766300 -1.34014200 1.14343900
H 7.01091900 -2.92325000 0.99553600
H 8.40585500 -2.75471400 2.14170300
